# The Actin‐Binding Prolyl‐Isomerase Par17 Sustains Its Substrate Selectivity by Interdomain Allostery

**DOI:** 10.1002/prot.26807

**Published:** 2025-03-12

**Authors:** Anna Sternberg, Jennifer Lynne Borger, Mathilda Thies, Anja Matena, Mike Blueggel, Bianca E. Kamba, Christine Beuck, Farnusch Kaschani, Markus Kaiser, Peter Bayer

**Affiliations:** ^1^ Structural and Medicinal Biochemistry, Center for Medical Biotechnology (ZMB) University of Duisburg‐Essen Essen Germany; ^2^ Quality Management Consultant at validAID Bonn Germany; ^3^ TUM, Bioverfahrenstechnik Straubing Germany; ^4^ Consultant Sustainability Services München Germany; ^5^ Chemical Biology, Center for Medical Biotechnology (ZMB) University of Duisburg‐Essen Essen Germany; ^6^ Analytics Core Facility Essen (ACE), Center for Medical Biotechnology (ZMB) University of Duisburg‐Essen Essen Germany

## Abstract

The human peptidyl‐prolyl‐*cis*/*trans* isomerases (PPIases), Parvulin 14 and Parvulin 17, accelerate the *cis*/*trans* isomerization of Xaa‐Pro moieties within protein sequences. By modulating the respective binding interfaces of their target proteins, they play a crucial role in determining the fate of their substrates within the cell. Although both enzymes share the same amino acid sequence, they have different cellular functions. This difference is due to a 25 residue N‐terminal extension present in Par17 but absent in Par14. Using activity assays, NMR spectroscopy, and mass spectrometry, we demonstrate that the N‐terminal extension of Par17 determines substrate selectivity by an intramolecular allosteric mechanism and exhibits a target‐binding motif that interacts with actin.

## 1 Introduction: The N‐Terminus Determines Par17's Role in Cell‐Function

**FIGURE 1:**
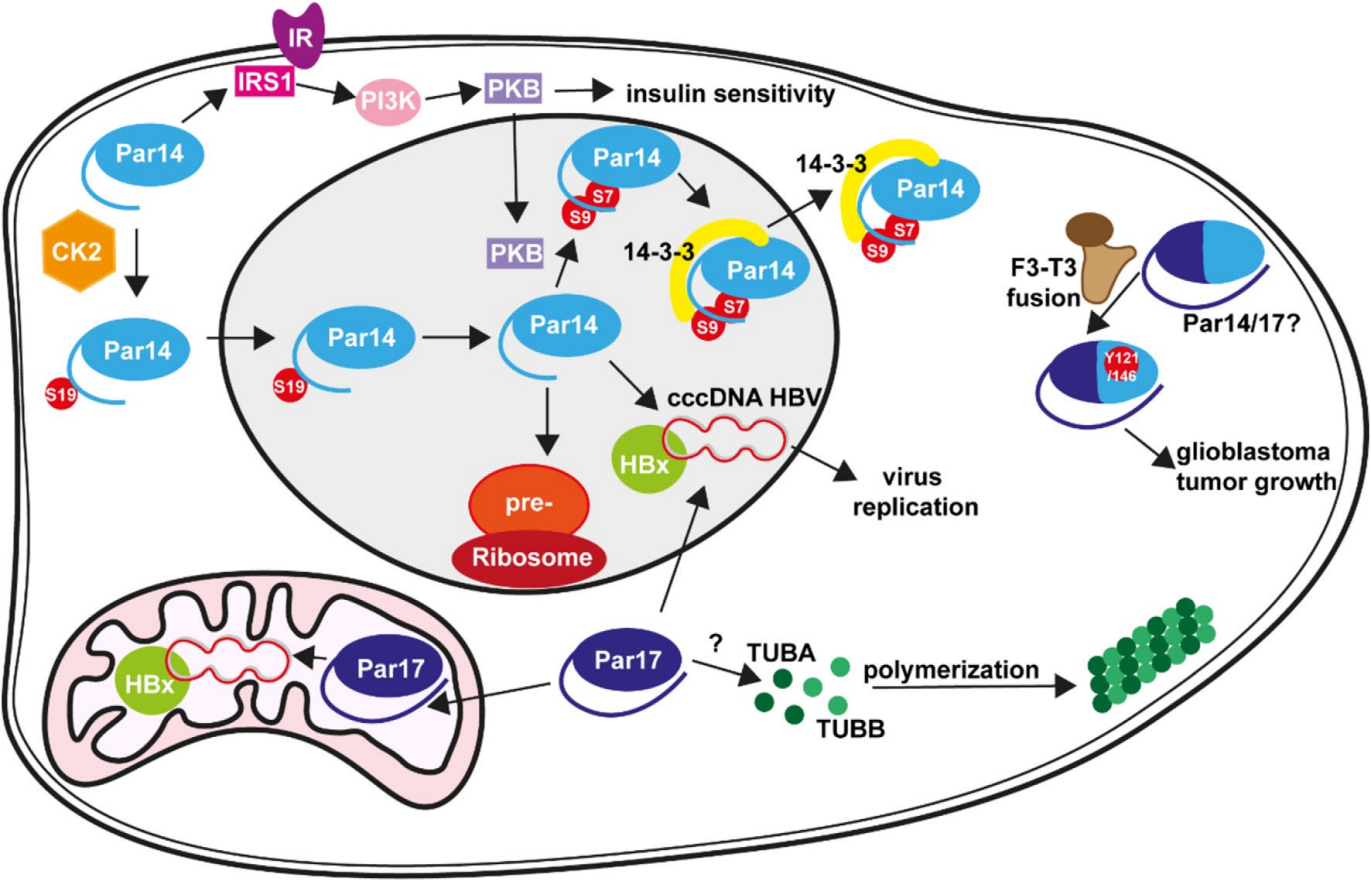
Schematic view of the cellular role of Par14 and Par17. Par14 (light blue) shuttles between the nucleus and the cytoplasm and is associated with insulin sensitivity, ribosomal biogenesis, rRNA processing, viral replication of HBV, and glioblastoma tumor growth. Par17 (dark blue) is transported into the mitochondrion and associated with tubulin polymerization, virus replication and glioblastoma tumor growth [12, 13, 14, 15, 16, 17, 18, 19, 20]. CK2 = casein kinase 2, 14–3‐3 = 14–3‐3 protein; IRS‐1 = insulin receptor substrate 1; IR: Insulin receptor, PI3K = phosphatidylinositol‐4,5‐bisphosphate 3‐kinase, PKB = phosphokinase B, TUBA, TUBB = tubulin; HBx. Cytoplasm (white), nucleoplasm (gray), and mitochondrion (pink) are colored.

Human cells express two distinct types of peptidyl‐prolyl *cis/trans* isomerases of the parvulin family—Pin1 and Par14/17 [1, 2, 3, 4]. Pin1, the only human parvulin that isomerizes pSer/pThr‐Pro sequence motifs in target proteins, is a two‐domain protein with a WW‐domain at the N‐terminal of its catalytic entity. Pin1's various functional roles [5, 6] emanate from an interdomain interaction [7, 8], which allosterically modulates the PPIase activity, as well as from post‐translationally controlled target binding affinity [9]. Pin1 is predominantly found in the nucleus of the cell. On the other hand, Par14 and Par17 are expressed by alternative transcriptional initiation from the PIN4 gene located on the X‐chromosome [2]. Both enzymes isomerize target proteins at Xaa‐Pro motifs, where Xaa is a non‐phosphorylated residue preceding proline [4, 10, 11]. Par14 and Par17 share an identical amino acid sequence, including a flexible N‐terminus and a catalytic domain, but differ in an N‐terminal extension of 25 amino acids present in Par17 and absent in Par14. Both proteins occur in the cytoplasm but are translocated to different compartments within the cell (Figure 1). Par14 is mainly located in the nucleus [19], concentrating in the nucleolus during interphase [13, 21]. In contrast, Par17 resides within the mitochondrion [22], but was also identified in the sub‐cellular fraction of the cell membrane [10, 22].

Although Par14 has a low molecular weight, its import to the nucleus is controlled by its N‐terminus. Specifically, the residues Ser^7^–Lys^14^ function as a nuclear localization signal (NLS) [19]. This import process is also modulated by the phosphorylation of Ser^19^ by casein kinase 2 (CDK2) [16]. The protein 14‐3‐3 orchestrates nuclear export depending on the simultaneous phosphorylation of Ser^7^ and Ser^9^ [15]. The import of Par17 into the mitochondrion depends on the N‐terminal 25 residue extension [22], which is predicted to exhibit a mitochondrial targeting signal. Further transport into the inner mitochondrial membrane is facilitated by the mitochondrial membrane potential.

The different localization patterns of Par14 and Par17 correlate with different functionalities of the proteins. Par14 associates with the pre‐ribosomal ribonucleoprotein (pre‐rRNP) complex [13, 21, 23] and chromatin in vivo [24]. The N‐terminus of Par14 is responsible for high‐affinity DNA‐binding [4, 19, 22] and mandatory for association with the pre‐RNP complex [13]. Since a reduced amount of Par14 in cells slowed down the processing of pre‐rRNA to 18 and 28S rRNAs, Par14 was attributed to a role in rRNA processing. In the cytoplasm, Par14 was found to associate with insulin receptor substrate 1 (IRS1) [20], in an N‐terminal depending manner, leading to enhanced phosphorylation of IRS1 as well as downstream phosphoinositide 3‐kinases (PI3K) binding and protein kinase B (PKB) phosphorylation. Additionally, Par14 is involved in the peroxisome biogenesis complex in FGFR3–TACC3 gene fusion cancer cells [12].

Only a few have focused on Par17. The enzyme catalyzed tubulin polymerization in vitro in a GTP and concentration‐dependent manner [10, 25]. For efficient polymerization, the presence of intact PPIase activity and the N‐terminus were required. The tubulin binding sites of Par17 were narrowed down to the first α‐helix (Lys^75^–Lys^85^) and an area including the second βsheet‐(Gln^103^–Phe^118^). In addition, Par17 was found to interact with proteins involved in cellular transport and cell motility and it was shown to polymerize actin in vitro [26]. The N‐terminus and catalytic activity were essential for this polymerization reaction. When Par17 was knocked down in cells, it had a pronounced negative effect on their motility and affected their migration capability [26]. Owing to its mitochondrial localization, Par17 also affiliates with proteins involved in β‐oxidation and oxidative phosphorylation [26].

Recently, Saeed and coworkers discovered [17, 18] a previously unrecognized function of Par14 and Par17 in virus replication [27]. In the presence of HBx protein (HBx), both isoforms upregulated hepatitis B virus (HBV) replication through binding to HBx and the cccDNA. Mutants of the isoforms, characterized by single amino acid exchanges like S19/44A (preventing nuclear import) and D74/99A (decreasing isomerization efficiency), lost the effect on viral replication.

According to the Protein Atlas (HPA), Pin4 mRNA can be found in almost all tissues [28]. However, distinguishing between the two isoforms of Par17 and Par14 in tissues or cell cultures is technically difficult. As a result, many studies have not been able to separate the contributions of each of the two isoforms to a specific cellular event. In addition, the impact of the N‐terminal 25 amino acids on the enzymatic capacity is insufficiently explored, although a recent study attempted to address this issue [29]. Since the N‐terminus of Par14/17, like the WW of Pin1, is believed to determine function, localization, and target recognition, we aimed to investigate the role of the N‐terminus in Par14 and Par17 on target binding and catalytic activity.

## 2 Results

### 2.1 Par17 Features Higher Substrate Selectivity Than Par14 and the Catalytic Core Domain

**FIGURE 2:**
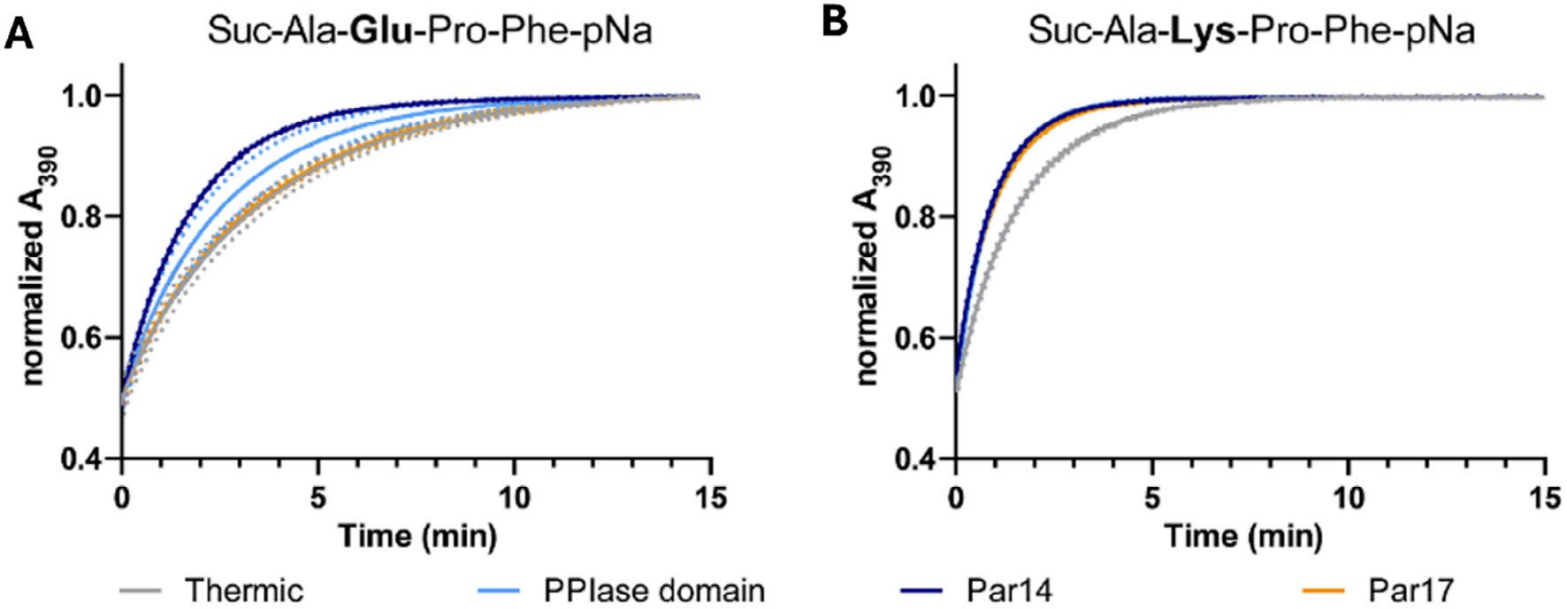
Protease‐coupled isomerase assay. Representative substrate peptides were measured in the absence (gray) or presence of Par17 (orange), Par14 (dark blue), and the catalytic domain (PPIase) (light blue). Changes in absorbance over time for model substrate (A) Suc–Ala–Glu–Pro–Phe–pNa and (B) Suc‐Ala‐Lys‐Pro‐Phe‐pNa are depicted. Dots represent the standard deviation of at least three measured replicates (for the exact number of measurements see Table S1).

To determine the catalytic efficiency and substrate specificity of Par14 and Par17, we performed a protease‐coupled assay [30]. In this assay, the *trans* form of a *para*‐nitroaniline (pNA) tagged model substrate is cleaved by chymotrypsin, and the resulting change in absorption at 390 nm due to free pNA is measured. When the model substrate is added to the protease‐containing buffer, all *trans‐Xaa‐Pro* isomers are immediately cleaved by chymotrypsin. Therefore, the extent of the first signal burst observed in the absorption spectrum correlates with the amount of *trans* isomers of the model substrate formed during the experimental dead time. The remaining *cis*‐Xaa substrates now start to isomerize by thermal (in the absence of PPIase) or catalytic reaction (in the presence of PPIase). The newly formed *trans*‐Xaa substrates are immediately cleaved, and isomerization can thus be monitored over time (Figure 2). Eighteen different substrate peptides with varying amino acids at the Xaa position (Suc‐Ala‐**Xaa**‐Pro‐Phe‐*p*Na) were measured to determine the isomerase activity and substrate selectivity of Par14 (Par17_Δ1–25_), Par17, and the catalytic domain (Par17_Δ1–60_) (Table S1). The catalytic efficiency is obtained from the observed isomerization curves by calculating the difference between the rate constant in the presence of enzyme and the rate constant obtained from thermal isomerization.

**FIGURE 3:**
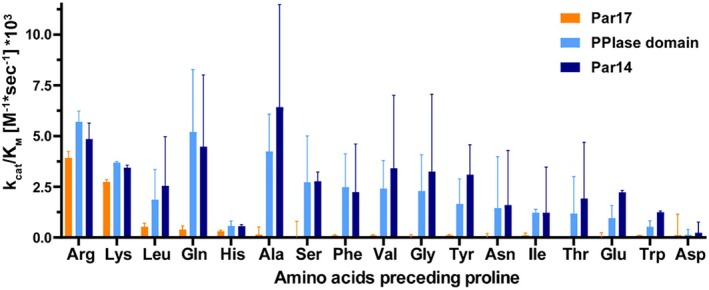
Substrate specificity of parvulins. The catalytic efficiency of Par17 (orange), of the isolated catalytic PPIase domain (light blue), and Par14 (dark blue) with various substrate peptides.

The *k*_cat_/*K*_M_ values of the parvulin isoforms and the catalytic domain varied within a range of 10^2^–10^3^ M^−1^ s^−1^ (Figure 3). Despite the high standard deviations measured, highest specificities in case of Par14 and the isolated catalytic domain were observed for the four substrates **Ala**‐Pro (6.4 × 10^3^ M^−1^ s^−1^, Par14), **Gln**‐Pro (5.7 × 10^3^ M^−1^ s^−1^, PPIase domain) as well as for **Arg**‐Pro (5.7 × 10^3^ M^−1^ s^−1^, PPIase domain) and **Lys**‐Pro (3.7 × 10^3^ M^−1^ s^−1^, PPIase domain). In general, both proteins showed catalytic efficiencies in the same order of magnitude for almost all measured substrate peptides. In contrast, *k*_cat_/*K*_M_ values extending 10^2^ M^−1^ s^−1^ were found only for five substrate peptides when examining Par17. The *k*_cat_/*K*_M_ values measured against substrates exhibiting **Arg**‐Pro (3.9 × 10^3^ M^−1^ s^−1^) and **Lys**‐Pro (2.7 × 10^3^ M^−1^ s^−1^) moieties were approximately 10 times higher than those observed for peptides carrying **Leu**‐Pro, **Gln**‐Pro or **His**‐Pro motives. Moreover, the specificity of Par17 against the positively charged substrates is about 30 times higher than for the remaining substrate peptides.

In conclusion, Par17 is more selective for positively charged residues such as Arg and Lys preceding proline and strongly discriminates these species against all others, while the shorter Par14 isoform and the isolated catalytic domain generally do not [11]. In addition, Par17 shows higher specificity against positively charged residues preceding proline in terms of higher *k*_cat_/*K*_M_ values concerning all other tested model substrates. As the parvulin isomers only differ in their N‐terminal sequences, it can be assumed that the 25‐residue extension of Par17 induces this high substrate selectivity/specificity probably by interacting with its catalytic domain and acting as an allosteric modulator. To elucidate the origin of Par17's substrate selectivity, we followed this hypothesis and investigated the putative interaction of the core domains of Par14 and Par17 and their flexible N‐termini in more detail. The N‐terminal region within hPar17 is highly flexible [31]. Nevertheless, secondary structure predictions point towards an amphipathic α‐helical region [22]. Owing to its amphipathic character the helical region of hPar14 is nascent in an aqueous solution and is predicted to be stabilized upon binding to targets.

### 2.2 Nuclear Magnetic Resonance (NMR) Chemical Shift Perturbation Experiments Suggest an Intra‐Molecular Interaction of the N‐Terminus and Parvulin Core Domain

For a few PPIases, such as the human FKBP38 (2013 Yoon) and FKBP42 from *Arabidopsis thaliana* [32] an interaction of a flexible N‐terminus with its catalytic domain was observed in previous studies. If the N‐termini of Par14 or Par17 comparably interact with their catalytic domains, the chemical environment of residues forming the binding epitopes would be affected. This, in turn, can be monitored by chemical shift changes of ^1^H‐^15^N‐signals in NMR spectra when comparing different truncation constructs or isoforms of the protein of interest. Concerning Par14/17 such changes in the chemical environment, are caused by either structural differences between the constructs (position of the N‐terminus due to construct length and the associated electrostatic changes) or by interaction of the catalytic domain and its N‐terminus. When comparing the HSQC spectra of Par14 and Par17 Weiwad and coworkers [25] identified minor chemical shift changes in amide resonances of residues residing in the region of Lys^100^ to Ala^125^ (Par17), which might indicate differences in the spatial arrangement of the N‐termini relative to their catalytic domains. However, in the above‐mentioned study, the spectra were recorded and compared under disparate buffer conditions.

**FIGURE 4:**
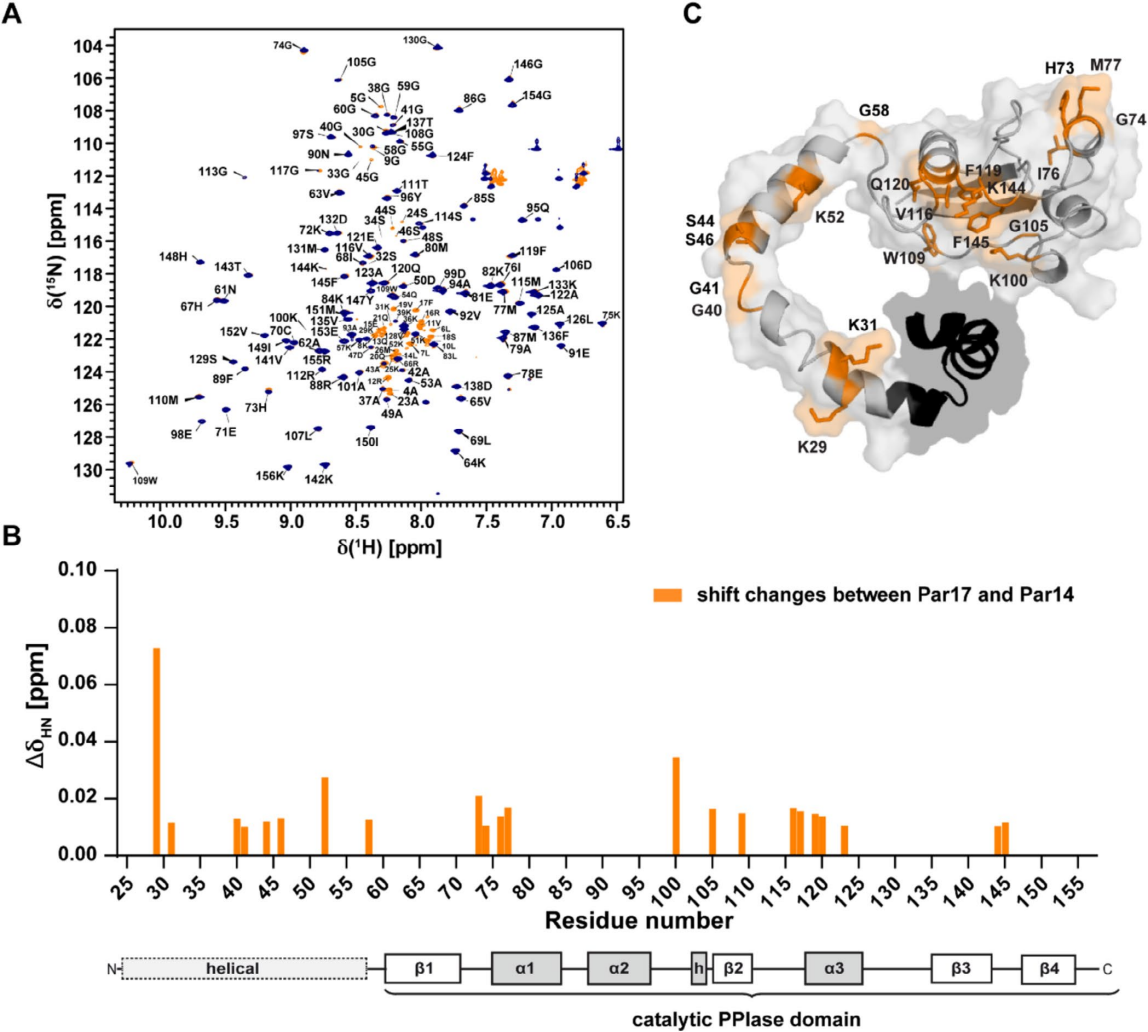
Deviations in chemical shifts within ^1^H‐^15^N‐HSQC spectra of Par14 and Par17. (A) Overlay of ^1^H‐^15^N‐HSQC spectra of Par14 (dark blue) and Par17 (orange). Amide resonances were assigned and labeled according to Sekerina et al. [34] Lin et al. [31]. Measurements were carried out at 25°C in 50 mM KPi buffer at pH 6.4. (B) Chemical shift differences occurring between corresponding amide resonances in Par14 and Par17 were plotted against the residue numbers (Par17 nomenclature). The secondary structure of both parvulins starting with residue 25 (Par17) is shown at the bottom of the graph. α‐helices (α) and helical turns (h) are depicted as gray boxes and β‐sheets (β) as white boxes. The I‐TASSER software handles the N‐terminal sequence (dotted box) as α‐helical region. (C) Chemical shift differences between resonances of residues within Par14/Par17 (orange) depicted on the surface of Par17 (calculated by I‐TASSER based on the structure of the PPIase domain PDB: 3UI6). The first 25 N‐terminal amino acids, unique to Par17, are displayed in black.

To further inspect the observation reported by Weiwad and coworkers, we recorded ^1^H‐^15^N‐HSCQ spectra of Par14 and Par17 under identical conditions and compared the chemical shifts of the corresponding amide resonances (Figure 4A and Table S2). Weak but significant deviations in chemical shifts of resonances of residues within the N‐terminus could be assigned to Lys^29^, Lys^31^, Gly^40^, Ser^44^, Ser^46^, Lys^52^, and Gly^58^ (Par17 nomenclature). In addition, chemical shifts of resonances in residues along helix α_1_‐His^73^, Gly^74^, Ile^76^, and Met^77^ and within the catalytic domain such as Val^116^, Phe^119^, Lys^144^, and Phe^145^, which contribute to substrate binding [33], were affected, too (Figure 4B). The deviations in chemical shifts between Par14 and Par17 were mapped on a structural model of Par17 (Figure 4C).

The deviations in chemical shifts between resonances in the HSQC spectra of Par14 and Par17 suggest a different structural arrangement of the N‐termini within the parvulin isomers. Moreover, the simultaneous but diverse effect on resonances within the catalytic core domain assumes an interference of N‐terminal and core residues. On basis of chemical shift perturbation experiments Weiwad and co‐workers suggested the N‐terminal residues Lys^8^ and Gly^9^, along with Phe^17^ of Par17, as probable candidates for interacting with the core residues of the PPIase domain [25]. Our studies now suppose the participation of a broader number of N‐terminal residues in a putative interface formation. In terms of a stable complex, these residues should be identifiable using NOE data from NMR experiments. However, no long‐range NOE signals were extractable in NOESY spectra of Par17 [25] that could narrow down the interface regions between the catalytic domain and N‐terminus. Such discrepancies between chemical shift and NOE data can be interpreted in terms of a weak and transient interaction of the N‐terminal region and its catalytic domain.

### 2.3 Chemical Cross‐Linking Confirms Spatial Vicinity of the N‐Terminus and the Catalytic Core Domain

To detect putative transient binding of the N‐terminal region and its catalytic domain, cross‐linkers were used, which covalently fix such interactions making them detectable by subsequent mass spectrometry analysis. disuccinimidyl sulfoxide (DSSO) and photo‐reactive amino acids were used to capture these intramolecular interactions within Par14 and Par17 (M&M 1.1.3/1.1.5.3). The resulting complexes were separated by size exclusion chromatography (Figure S1) and the isolated monomeric intramolecular cross‐linked species were analyzed using LC–MS/MS. All cross‐links identified were listed in Table S4.

Cross‐links could be identified between residues within the catalytic domain and between amino acids of the catalytic domain and the N‐terminus for both parvulin isoforms within all experimental approaches (Figure S1E,F). Cross‐links between residues within the N‐terminus have only been detected in DSSO experiments (Figure S1E). In detail, the first α‐helix at residue Lys^57^/Lys^82^ (Par14/Par17) as well as the loop region between α_2_ and β_2_ at Lys^75^/Lys^100^ (Par14/Par17) of the catalytic domain were cross‐linked to amino acids within the N‐terminus (Lys^14^/Lys^39^; Par14/Par17). Further linkages were detected between the N‐terminal residues Lys^26^ and Lys^108^ in Par14, as well as Lys^31^/Lys^36^ and Thr^143^/Lys^144^ in Par17. These core residues reside within the flexible, extended loop of the catalytic domain, leading into the fourth β‐sheet (Thr^143^ to Gly^146^). Cross‐link studies with photo amino acids (Figure S1F) revealed a spatial orientation of the first 20 N‐terminal amino acids close to the end of the β_2_‐region (Met^85^) as well as to the h‐β_3_ loop (Met^106^) in Par14. Similarly, the experiment with Par17 proposes close proximity of Ser^32^ and Lys^36^ (Par17) to residues in helix α1 (Lys^75^ to Lys^84^). Cross‐links unique for Par17, which were found within both experimental approaches (using either DSSO or photo‐reactive residues), occurred between the N‐terminal residues Met^1^, Met^3^, and Lys^8^ and the first α‐helix (Met^77^, Met^80^, and Lys^82^) of the catalytic domain.

**FIGURE 5:**
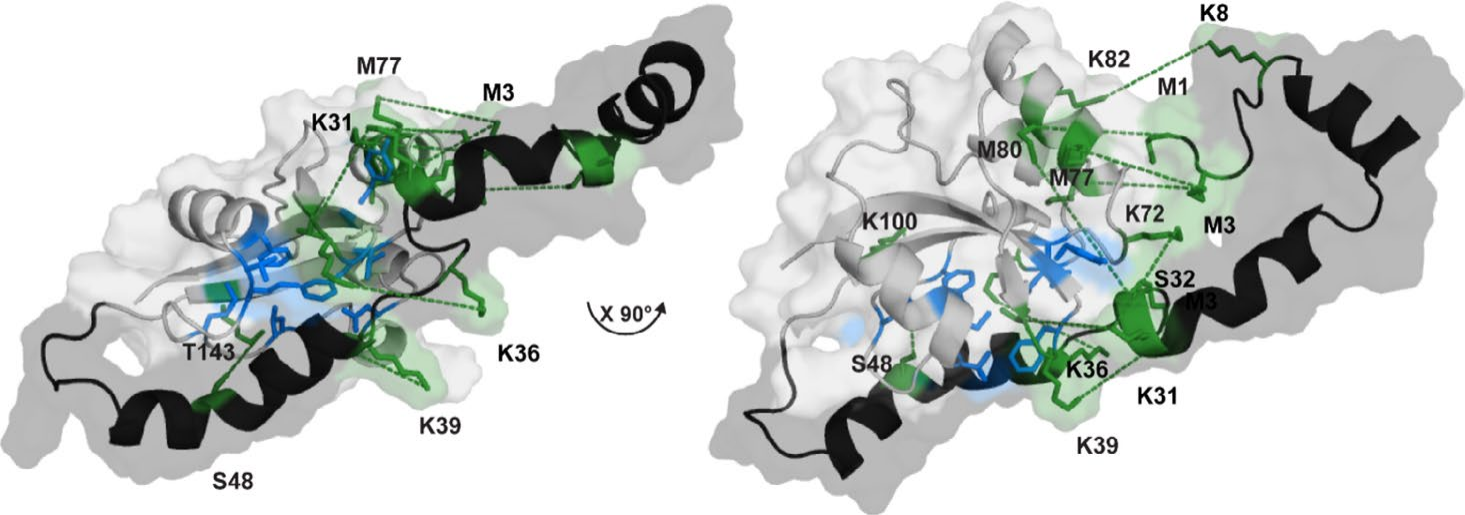
Constraint model of Par17. The model was calculated using the program YASARA and is based on the Par17 I‐TASSER model used in this work. The identified cross‐links were used as experimental restraints. Photo‐reactive cross‐links and DSSO cross‐links between the PPIase domain and the N‐terminus are illustrated as dashed lines, with the specific residues involved highlighted in green and annotated with their respective residue names. The amino acids involved in the catalytic process are marked in blue.

To investigate this putative interaction of the catalytic PPIase domain and the N‐terminal extension, a more advanced structural model based on the former I‐TASSER model of Par17 (Figure 5) was calculated using the program YASARA by embedding the experimental cross‐links between the N‐terminus and the PPIase domain as distance restraints. After energy minimization, the model obtained illustrates the proposed spatial proximity between the first residues of the N‐terminus and the first α‐helix within the catalytic domain of Par17.

The YASARA model of Par17 can explain the NMR chemical shift differences within the resonances of helix α_1_ and residues of the active sides found when comparing Par14 and Par17 spectra. These deviations are caused by the spatial vicinity of helix α_1_ as well as of the active center to the very first residues in the N‐terminus of Par17 which is absent in Par14. The model manifests an intramolecular transmission of changes between two sites at a distant residing in different domains [35]. Although we have to be aware of the fact, that the structural model may represent a preferred and favored transient interaction mode, this putative intramolecular interference of the N‐terminus and the PPIase domain in Par17 may cause an allosteric effect and thus explains the different enzymatic behavior of Par17 and Par14.

### 2.4 Higher Selectivity for Arg‐Pro/Lys‐Pro Peptides Correlates to Higher Microscopic *K*_D_ Values

Although all parvulin isoforms exhibit a similar substrate specificity for Arg–Pro and Lys–Pro substrates, Par17 is more selective for these compounds when compared to the isolated catalytic domain or Par14. In addition, the spatial adjacency between residues of helix α_1_ within the catalytic domain and its very N‐terminal region in Par17 is absent within the shorter parvulin constructs. We therefore elucidated if the higher selectivity of Par17 originates from its intramolecular domain arrangement by studying the effect of substrate binding on Par17. We expected that differences in selectivity between peptides are reflected in different chemical shift patterns of the corresponding Par17‐substrate complexes.

**FIGURE 6:**
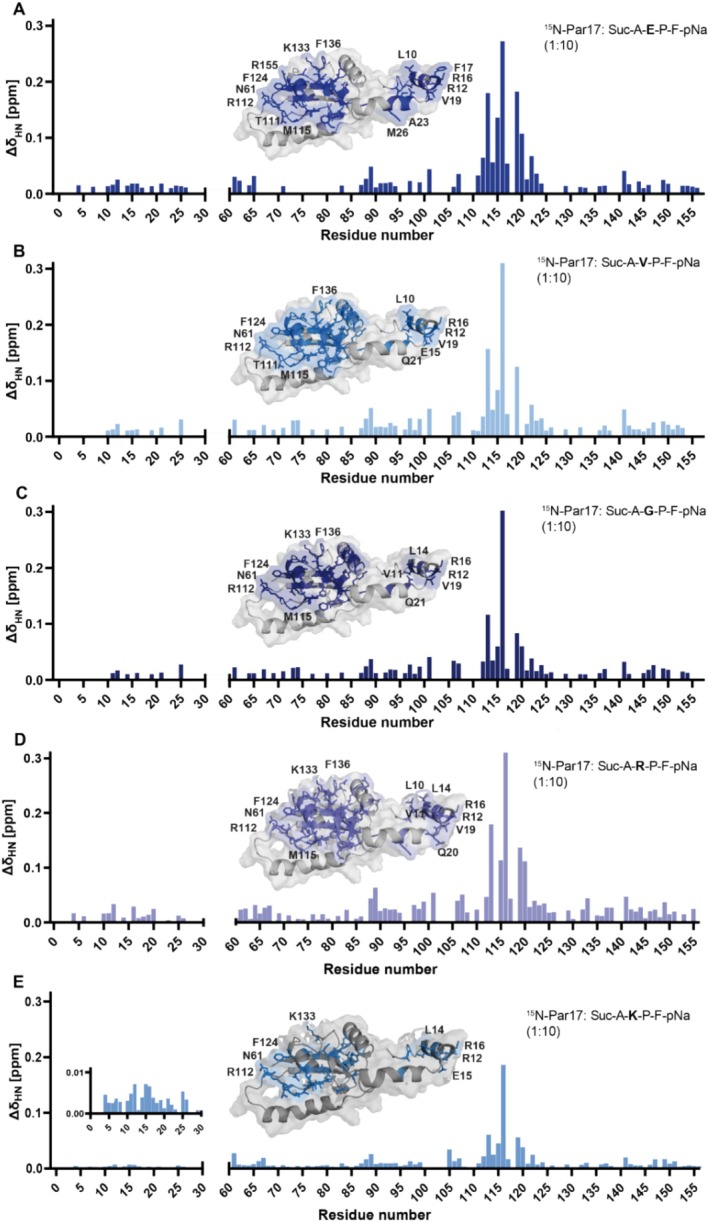
Shift changes in Par17 after the addition of representative model substrates. Chemical shift changes measured for amide resonances of Par17 before and after addition of the respective model substrates Suc‐Ala‐**Glu**‐Pro‐Phe‐pNa (A), Suc‐Ala‐**Val**‐Pro‐Phe‐pNa (B), Suc‐Ala‐**Gly**‐Pro‐Phe‐pNa (C), Suc‐Ala‐**Arg**‐Pro‐Phe‐pNa (D) and Suc‐Ala‐**Lys**‐Pro‐Phe‐pNa (E) (Par17: 0,3 mM, substrates: 3 mM). Shift changes were calculated from the overlaid ^15^N‐BEST‐TROSY‐HSQC spectra recorded at 20°C in 50 mM KPi buffer at pH 6.7 at a ratio of protein to substrate of 1:10. All residues whose amide signals underwent chemical shift changes (> 0.01 ppm) were highlighted. For titration of Suc–Ala–Lys–Pro–Phe–pNA (E) shift changes > 0.004 ppm were highlighted. The respective shift changes are highlighted (blue) on the calculated YASARA structure of Par17, with selected amino acids labeled using PyMOL.

To test this hypothesis, representative model substrates exhibiting residues at the Xaa position that were either high selectively catalyzed, such as Lys and Arg, or low selectively catalyzed such as Glu, Val, and Gly were added to ^15^N‐labeled Par17. BEST‐TROSY‐HSQC spectra were recorded in the absence and presence of various peptide concentrations. The amide signals were assigned and combined chemical shift changes between the signals of free and substrate‐bound isomers were calculated. The resulting shift differences were plotted against the residue numbers of Par17 (Figure 6).

In the presence of each model substrate, the highest shift changes arise within resonances representing amino acids of the catalytic domain. Residues of helix α_2_ and the adjacent loop (Met^87^ to Ala^101^) as well as Thr^111^ to Phe^124^ and a region at the C‐terminus are affected in all experiments. This observation is in agreement with the binding of the substrates to the active center of the catalytic domain. In general, no striking differences in the binding of peptides to the catalytic domain of Par17 are found concerning chemical shift patterns when comparing peptides catalyzed with low and high catalytic efficiency. This means that catalytic selectivity is not reflected in the chemical shift data observed for Par17 upon substrate binding. Next, we performed titration experiments to reveal if binding of the Glu–Pro, Val–Pro, and Gly–Pro substrates is attended by substantial differences in microscopic (residue‐specific) *K*_D_ values (Figure S2 and Table S3) when compared to Arg–Pro or Lys–Pro peptides. The residue‐specific *K*_D_s obtained from titration curves of 116 V of Arg‐ and Lys‐peptides are two to three times increased (17 and 24 mM, respectively) concerning the corresponding K_D_ values of all other tested peptides (6 and 7 mM, respectively). In contrast, the *K*_D_ values obtained from titration data of other residues near the binding site are in the range of 4–9 mM in all tested peptides. Although a higher selectivity for Arg–Pro and Lys–Pro peptides correlates to higher *K*_D_ values with respect to residue 116 V, the very low binding affinities made it difficult to obtain final and conclusive data implications from these values.

### 2.5 The N‐Terminus of Par17 Exhibits a Target‐Binding Epitope

In addition to the chemical shift changes observed within the catalytic domain, changes within amide resonances occur along a stretch of N‐terminal residues (Ala^4^ to Lys^25^) in the presence of all substrates investigated (Figure 6). These changes may result either from a substrate‐induced loss of the transient intradomain interaction or from an anchorage of the peptides to binding epitope at the N‐terminus (or both). Changes in chemical shifts of resonances of residues residing in sequentially distant epitopes were previously observed upon addition of substrate to the structurally related parvulin PPIase hPin1 [8] and resulted from two different pSer/Thr binding sites, one located in the catalytic domain and the other in the preceding N‐terminal WW domain. Additionally, Burgardt and co‐workers reported a specific Calmodulin binding site within the N‐terminus of Par17 [25].

**FIGURE 7:**
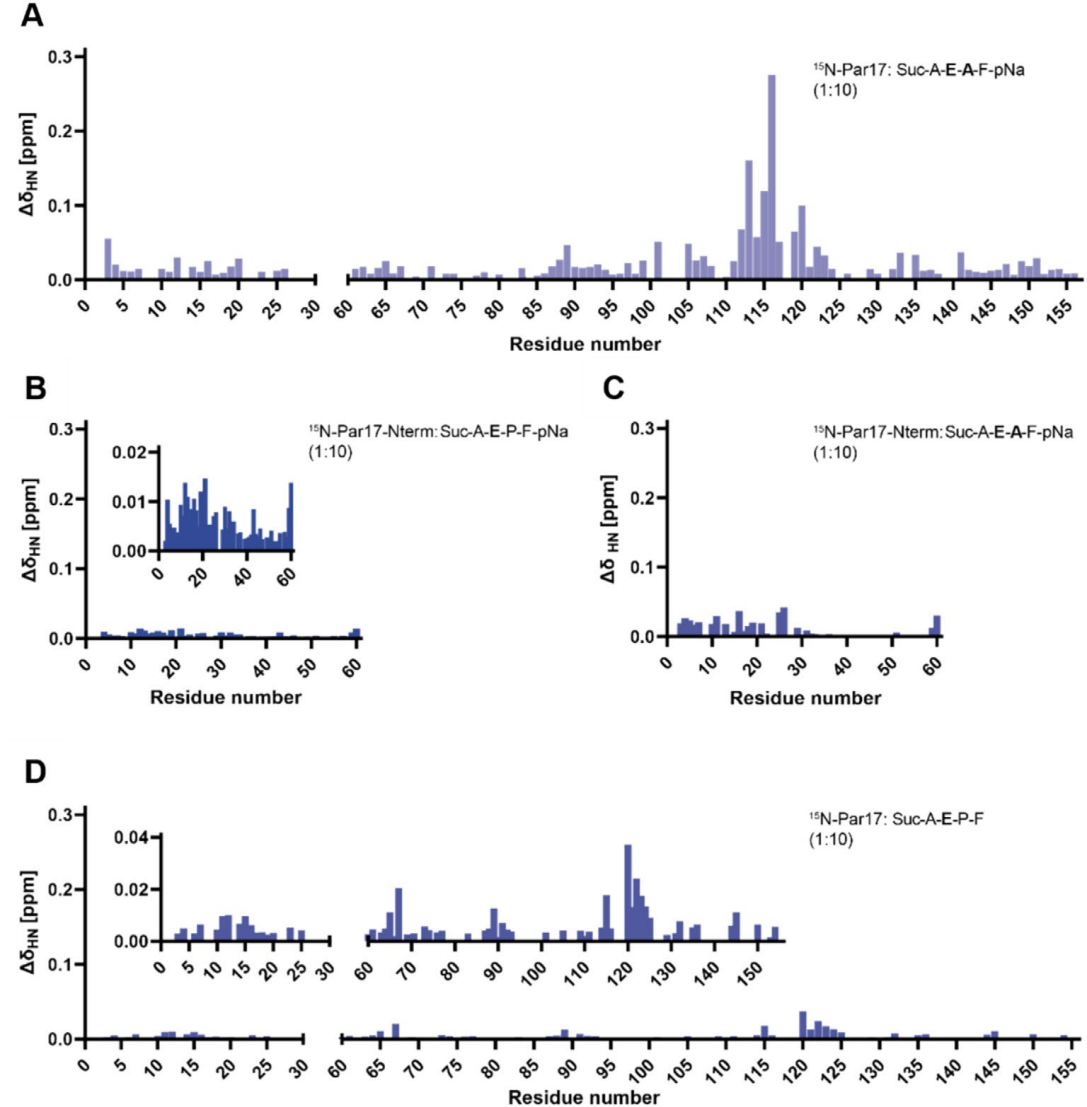
Shift changes in Par17 and Par17‐N‐term after the addition of different glutamate‐containing substrates or pNA. Chemical shift changes measured for amide resonances of Par17 before and after addition of the respective model substrates Suc–Ala–**Glu–Ala**–Phe–pNa (A), Suc–Ala–**Glu–Pro**–Phe–pNa to the N‐terminus of Par17 (B), Suc–Ala–**Glu–Ala**–Phe–pNa to the N‐terminus of Par17 (C), Suc–Ala–**Glu–Pro**–Phe (D) (Par17: 0.3 mM, substrates: 3 mM). Shift changes were calculated from the overlaid ^15^N‐BEST‐TROSY‐HSQC spectra recorded at 20°C in 50 mM KPi buffer at pH 6.7.

To elucidate the physical nature of the chemical shift changes in Par17 upon the addition of selected substrate peptides, we investigated whether the isolated N‐terminus of Par17 (Par17_Δ61–156_) is also capable of binding various substrates on its own. The titration experiment using the representative substrate peptide Suc–Ala–Glu–Pro–Phe–pNA and ^15^N‐labeled Par17_Δ61–156_ revealed a similar pattern of chemical shift changes as obtained in the presence of full‐length Par17 (Figure 7B). This indicates the existence of an independent binding epitope located within the N‐terminus.

In the next step, we studied if this binding epitope is selectively interacting with peptides carrying Xaa‐Pro motifs. We already knew from former studies that model peptides carrying alanine instead of proline in the Xaa position are capable of binding to the hydrophobic catalytic center without being catalyzed [36]. Studies with the substrate Suc–Ala–Glu–Ala–Phe–pNA confirmed that such proline‐free peptides interact with the N‐terminal epitope, too (Figure 7C), thereby inducing similar chemical shift patterns as Xaa‐Pro peptides did. In closer investigation, the peptide binding epitope overlaps with the N‐terminal intra‐molecular interface defined by cross‐linking studies and suggested by the YASARA models.

### 2.6 Functional Role of Par17 N‐Terminus in Actin Binding

In former mass spectrometry studies, Actin was found to be an interactor of Par17 [26]. Moreover, when comparing the ^1^H‐^15^N‐HSQC spectrum of ^15^N‐labeled Par17 in the absence and presence of 45 μM non‐muscle actin, changes in the peak intensities of amide resonances from Arg^66^, Leu^84^, Lys^100^, and Ile^149^ as well as in resonances of the 25 N‐terminal residues were found [26]. Both experimental studies indicated a putative actin‐binding epitope of Par17. To shed light on the mechanism of action of Par17 and its N‐terminus, we studied the interaction of the protein with this cellular target protein.

**FIGURE 8:**
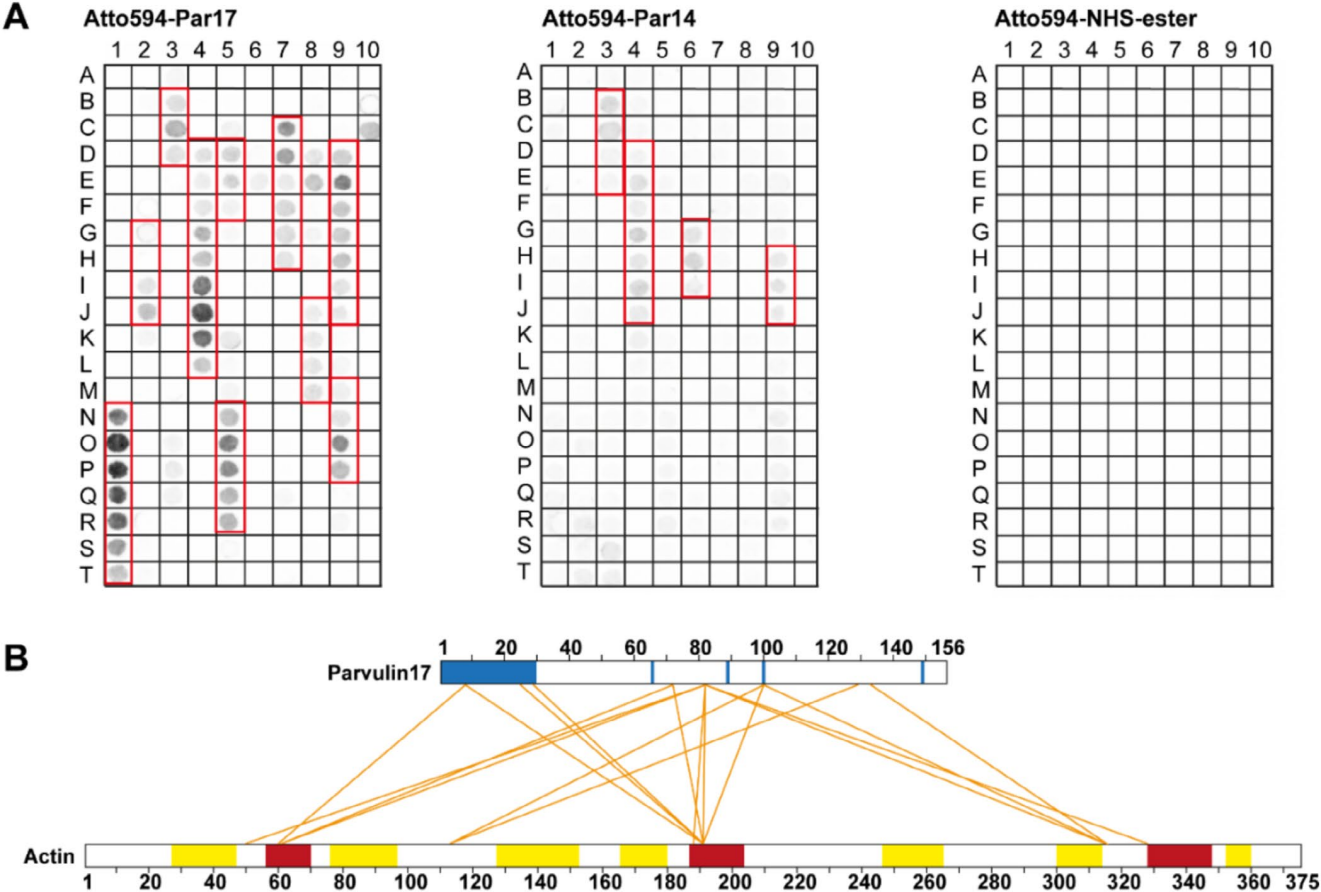
Defining the binding sides within Par17 and Actin using peptide spotted array and cross‐linking. (A) Peptide array with 12‐mers of β‐Actin amino acids (peptide walk: 2 Amino acids per step) incubated with Atto594‐Par17 (left), Atto594‐Par14 (middle) or the unpaired Atto594‐NHS‐ester (right). Putative binding sides on Actin occur as blackened‐filled circles. The level of blackening correlates with the strength of binding. For the Atto594‐labeled parvulins all sequences with a length equal to or greater than three were classified as possible binding sites. (B) Schematic representation of the DSSO cross‐links between Par17 and Actin (orange lines depict an identified connection between the two proteins). Crosslinks found with a score higher than 130 [37] are shown. The amino acids of Par17 with reduced intensities in the presence of Actin (blue) identified by NMR by Goehring and coworkers [26] and the putative Actin‐binding regions of Par17 identified by the peptide array (yellow) are highlighted. Red segments: Actin sites which overlap with the putative binding sites from the peptide array experiment.

In the first step, a peptide array was designed based on the amino acid sequence of actin (Figure 8). This array consisted of a library of overlapping peptides (12‐mers), each shifted by 2 amino acids across the entire sequence of β‐actin (aa 1–375), immobilized repeatedly on cellulose membranes. We imaged the binding of Atto‐labeled Par17 and Par14 (Figure 8A, left and middle blot) to these peptides. For Par14, four sequences were identified as possible target regions while10 segments were found for Par17 (Figure 8A, left and middle blot (red boxes), Table S6). The stretches Pro^27^–Lys^50^ (N1–T1), Tyr^53^–Pro^70^ (G2–J2), Glu^167^–Gly^182^ (D5–F5), Asp^187^–Arg^206^ (N5–R5), Gly^245^–Phe^266^ (C7–H7), Leu^299^–Glu^316^ (J8–M8) and Ile^345^–Tyr^362^ (M9–P9) are unique for Par17, whereas three of the actin binding sequences Glu^83^–Glu^100^ (B3–E3), Phe^127^–Gly^150^ (D4–J4) and Arg^335^–Ser^350^ (H9–J9) are shared by Par14 and Par17. A unique sequence observed for Par14 only ranges from Lys^213^–Ala^228^ (G6–I6). However, for Par17 the two epitopes around D4–J4 and H9–J9 are longer in sequence (Phe^127^–Asp^154^ (D4–L4) and Ile^327^–Ser^350^ (D9–J9)) showing more intensive spots (Figure 8A, left blot). These facts pinpoint a stronger binding of Par17 to certain sequences of the actin peptides and correlate with the former mass spectrometry studies [26]. Seven of the identified segments carried a Xaa‐Pro motif with varying amino acids at the Xaa position (Arg^37^–Pro^38^, Tyr^69^–Pro^70^, Ala^97^–Pro^98^, Trp^129^–Pro^130^, Lys^171^–Pro^172^, Cys^257^–Pro^258^, Tyr^306^–Pro^307^).

In a second step, Par17 and actin (non‐muscle actin (> 99% pure, 85% β‐actin/15% γ‐actin), Cytoskeleton Inc., Cat.#APHL99) were cross‐linked with DSSO (Figure S3). Cross‐links were detected and identified by mass spectrometry (Table S5) and depicted in a schematic 2D network (Figure 8B). Four interaction regions were identified on actin. Three of the actin sites overlap with the putative binding sites from the peptide array experiment (Figure 8B, red segments). The first region is located between Glu^57^–Pro^70^, the second at Gly^188^–Pro^204^ and the third comprises residues Ile^327^–Ser^350^. In addition, two interaction epitopes are observed on Par17. These regions comprise residues within the first 25 amino acids of the N‐terminus and amino acids located around the active center of Par17 between Lys^72^ and Lys^133^. The identification of these regions in Par17 as putative binding epitopes is supported by NMR binding studies of ^15^N‐labeled Par17 in the presence of non‐muscle actin (Figure 8B) [26].

**FIGURE 9:**
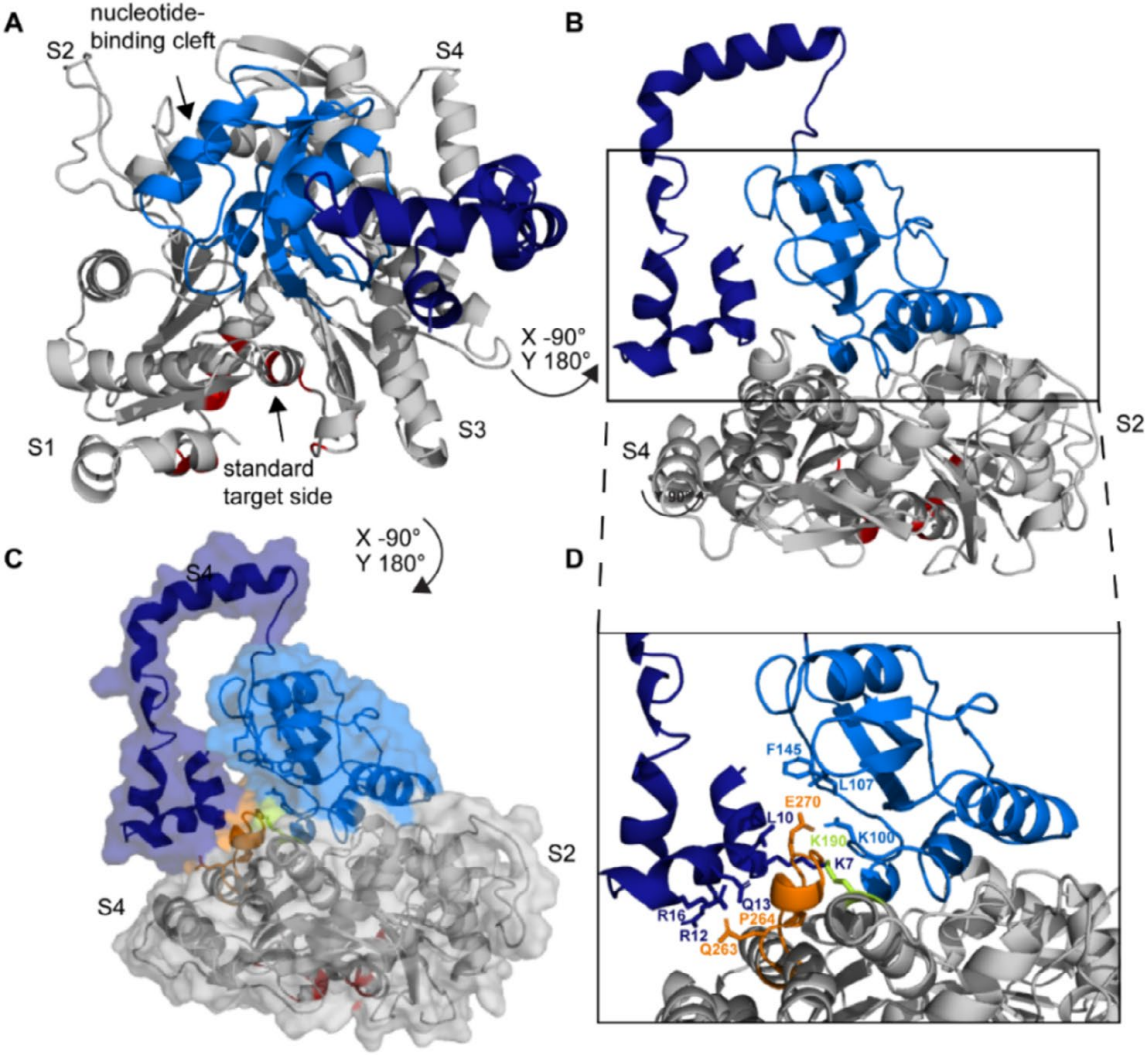
Interaction model of Par17 and β‐Actin. The interaction model between β‐Actin (PDB‐ID: 6ANU, Gray) and Par17 (PPIase domain: Light blue, N‐terminus: Dark blue) is depicted from different angles. The model was calculated by HADDOCK based on the presented experimental data. Energy minimization was performed with YASARA (Table S11). (A) The interaction model is shown from the top and the subunits of Actin and the standard binding site are labeled. (B) The interaction model depicted from the side and C: With the protein surface. (C, D) The loop of Actin that is enclosed by the N‐terminus and the PPIase domain of Par17 is highlighted (orange).

Subsequently, the experimental constraints from cross‐link, peptide array, and NMR studies were fitted into the program HADDOCK [38, 39] to calculate an interaction model of actin and Par17 by computational docking (Figure 9 and Table S10). Herein, the tertiary structure of actin consists of four subdomains (S1–S4) where two domains each are separated by a cleft [40, 41]. The cleft between S1 and S3 forms the standard target side of actin (Figure 9A, bottom; red color), while the cleft between S2 and S4 (Figure 9A, top) includes the typical ATP binding side. The catalytic domain of Par17 appears to bind to the actin interdomain interface where all four subdomains of actin merge (Figure 9A). This conforms to cross‐links at Lys^190^ (Figure 9C, green color) of actin (localized at the interface of the four subunits) to the PPIase domain of Par17. Par17's N‐terminus is oriented adjacent to its PPIase domain, both attaching the S3/S4 interface, (Figure 9A) and wrapping around the actin Phe^262^–His^275^ loop region (Figure 9C, orange color). Arg^12^, Gln^13^, and Arg^16^ of the putative target motif in Par17 are directly interacting with residues Gln^263^–Pro^264^ (Figure 9D) while Glu^270^ is in close contact with residues Lys^100^, Leu^107^, and Phe^145^ of the catalytic center of Par17.

Alternatively, we performed an Alphafold3 experiment, which generated a Par17‐actin complex that could not justify any of our experimental constraints. The binding of Par17 was found to occur on the opposite side of actin when compared to our HADDOCK model.

## 3 Discussion

We hypothesized that initially observed chemical shift differences in amide resonances within the ^15^N‐HSQC spectra of Par14 and Par17 point towards structural differences within the intramolecular arrangement of both enzymes. Cross‐link experiments and subsequent mass spectrometric analysis revealed a close vicinity of the 25 residue N‐terminal extension (residues Met^1^, Met^3^, and Lys^8^) of Par17 to amino acids of α‐helix 1 (Met^77^, Met^80^, and Lys^82^), which is located close to the active center of its catalytic domain. Concurrently, enzymatic studies of both PPIases recovered a higher selectivity of Par17 toward certain substrate targets, particularly peptides with positively charged amino acid within the isomerizable Xaa‐Pro moiety. This suggested an interdomain allosteric mechanism, and we related the chemical shift changes observed to an interaction of the N‐terminus and the PPIase domain. To confirm our interpretation, we used I‐TASSER and Haddock modeling with empirical data on Par17. The models pointed towards a steric interference between residues around the active center and those from the N‐terminal extension.

A similar weak interaction for a catalytic and its N‐terminal domain was also demonstrated by Kang and coworkers for the PPIase FKBP38 [42]. The presence of the N‐terminus resulted in chemical shift changes in residues of the catalytic domain and close to the putative isomerase active center of FKBP38. The presence of part of the N‐terminus resulted in inactivation of the PPIase domain, most likely caused by their interaction. Similarly, our data indicates that the N‐terminal extension causes higher selectivity toward Arg–Pro and Lys–Pro peptides. The presence of an intermolecular interface between N‐terminus and PPIase domain strongly suggests allosteric coupling within Par17. However, the absence of NOEs between resonances of both domains assumes that this allostery is dynamically rendered [35, 43].

Although we could not find a clear link between certain peptides' selectivity and distinct NMR chemical shift patterns of corresponding Par17‐substrate complexes, we did find that Arg–Pro and Lys–Pro peptides had higher microscopic *K*_D_ values when binding to the catalytic core domain of the different parvulin isoforms. Unfortunately, these higher microscopic *K*_D_ values measured at the high substrate and protein concentrations used in NMR experiments cannot instantaneously be translated into a physiologically meaningful signal without further experimental evidence. While our chemical shift data on substrate complexes eluded a simple quantitative interpretation in terms of intermolecular interaction of domains, they did allow us to detect a general peptide‐targeting interface within the N‐terminal extension of Par17. This binding motif resides within the stretch of Ala^4^ to Lys^25^ in the putative amphipathic α‐helix of the N‐terminus [22]. The fact that this extension is crucial for mitochondrial translocation and necessary for the polymerization of both, tubulin [10, 25] and actin [26], is consistent with the peptide binding motif's responsibility for the corresponding protein interactions. An affirmation of this assumption comes from our studies on the functional role of Par17. Using empirical data, our final model of actin and Par17 by computational docking strongly suggests a direct interaction of the N‐terminal extension with actin.

Actin‐binding proteins such as thymosin‐B4 [44], tropomodulin [45], or the β‐III‐spectrin actin‐binding domain [43] often have a long and flexible N‐terminus that wraps along the actin‐binding sites. According to our model, Par17's N‐terminus extends along the motif Gln^263^–Pro^264^. This hydrophobic plug of actin protrudes into the cleft between the N‐terminus and the PPIase domain, where the amino acid Glu^270^ enters the deepest. Glu^270^ is placed near the active center of the PPIase domain, close to residues Lys^100^, Leu^107^, and Phe^145^. Glu^270^ is involved in ADP‐Ribosylation of Actin by *Clostridium perfringens* Iota Toxin [46] and in the formation of an intermolecular iso‐peptide bond connecting actin monomers by *Vibrio cholerae* [47].

The hydrophobic plug between Gln^263^ to Glu^270^ [48] contains residues that are directly involved in interstrand contacts [49] to residues Arg^39^ and His^173^ of different actin monomer subunits (PDB ID: 6BNO). It is a prominent element maintaining the stability and health of the actin filament and sustains the monomer contact between protofilaments [50]. Missense mutations (Table S9) within this structural motif and the motifs covered by the intermolecular interface lead to various diseases [48, 50, 51]. The proper folding of the hydrophobic plug in actin is crucial for the formation of actin filaments. From our Par17/Actin complex model, we can retrieve a final mechanism that explains how hPar17 assists in the proper folding of the actin dimer interface. This may be facilitated by *cis*/*trans* isomerisation of either Tyr69–Pro90 of the sensory loop, Gln263–Pro264 of the hydrophobic plug or Arg37–Pro38 within the D‐loop. As the Arg37–Pro38 moiety fulfills the criterion for hPar17 substrate selectivity and its proper folding is critical for the formation of the lateral contact between Arg39/His40 and Gly268 of adjacent monomers (Table S8 and Figure S6), we suggest that native state *cis*/*trans*‐isomerisation of this element may accelerate actin polymerization as observed by Goehring et al. [52]. An alternative, thus complementary explanation could assume that the N‐terminal domain of hPar17 and the catalytic PPIase domain may act independently on two adjacent neighboring actin monomers. In this model, the PPIase interface would act on the D‐ and sensory loop regions while the N‐terminal helix may guide the hydrophobic plug of the adjacent actin subunit to the lateral contact side (Figures 9 and S7). Thus, our data allow explaining how Par17 could be involved in maintaining polymerization and healthy actin filament generation.

## 4 Conclusion

The human peptidyl‐prolyl‐cis/trans isomerases (PPIases), Parvulin 14 and Parvulin 17, modulate the binding interfaces of their target proteins. Although both enzymes share the same amino acid sequence, they were found to assist in different cellular events and are localized to different cellular compartments. This disparity is due to a 25 residue N‐terminal extension present in Par17 but absent in Par14.

We reported on the role of Par17's N‐terminus in determining the cellular function of the PPIase and demonstrated that the enzyme features higher substrate selectivity than Par14 for Arg–Pro/Lys–Pro containing target molecules.

We were made aware of this fact by NMR chemical shift perturbation experiments that pointed towards an intra‐molecular interaction of the N‐terminus and parvulin core domain. After confirming the spatial vicinity of the N‐terminus and the catalytic core domain by chemical cross‐linking, we found that this interaction lead to higher microscopic KD values which correlate with the higher selectivity of Arg–Pro/Lys–Pro moieties. As intra‐domain interaction influences the binding and selectivity of the enzyme, we concluded that the N terminal extension of Par17 determines substrate selectivity by an intramolecular allosteric mechanism.

Moreover, we provide evidence that the N‐terminus of Par17 exhibits a (proline‐independent) target‐binding epitope that contributes to Par17's enzymatic role in actin polymerization.

Our research forms a substantial incremental step in understanding how two proteins that are identical in their core catalytic domains but differ in a N‐terminal short extension can contribute to independent cellular processes.

## 5 Material and Methods

### 5.1 Cloning and DNA Transformation in *Escherichia coli*

Modified pET‐41 vectors (Novagen, Merck, Darmstadt, Germany) including an N‐terminal GST‐His6 fusion followed by a PreScission protease cleavage site, and the protein of interest [53] were used for expression of Par14, Par17, and the PPIase domain. Cloning of the Par17 N‐terminus (Par17_Δ61–156_) was achieved by inserting the corresponding DNA sequence into the modified pET41 vector of Par17 using primers 2572F (GAGAGACTGTTCCAGGGGCCCATGGC) and 2713R (TTGGTTCTCGAGCTATTAGCCACCACCTTTGGGACCTTGAGC) and a Pfu Plus Polymerase (Roboklon, Berlin, Germany) as prescribed by the manufacturer. For restriction, ApaI and XhoI (New England Biolabs, Frankfurt, Germany) were applied for 1 h at 37°C. All constructs were separated on agarose gels and purified with the NucleoSpin Gel and PCR Clean‐up kit (Macherey‐Nagel, Fisher Scientific, Schwerte, Germany) according to the manufacturer's description. Enzymes were inactivated at 65°C for 10 min. Cleaved constructs were ligated with T4 ligase (Metabion International AG, Planegg, Germany) for 1 h at 16°C. Transformation was performed with the head shock method. The competent *E. coli* strains (BL21 (DE3) T1r, Sigma‐Aldrich, Germany) were mixed with 50 ng plasmid DNA and incubated on ice for 20 min. Afterward the suspension was heat shocked at 42°C for 30 s and incubated on ice for a further 5 min. The cells were supplemented with SOC medium and incubated for 1 h at 37°C. Then the *E. coli* cells were either directly added to media for cultivation or grown on an agarose plate, each with required selective antibiotic.

### 5.2 Protein Expression and Purification

Transformed bacteria were grown in LB or M9 minimal medium (NMR experiments, photo cross‐linking) at 37°C. Expression was induced at an O.D._600_ of 0.8 by adding 200 μL of a 1 mM IPTG stock solution to 1 L of medium. Cells were incubated overnight at 30°C while shaking flasks at 160 rpm. After harvesting and cell lysis, the GST‐tagged proteins were purified from cell extract by affinity chromatography (washing buffer: PBS pH 7.5; high salt buffer: 400 mM NaCl_2_ in PBS; elution buffer: 20 mM glutathione in PBS). Protein fractions were concentrated, and GST fusion was cleaved by PreScission protease. To yield pure protein, size exclusion chromatography was performed (50 mM HEPES, 150 mM NaCl, pH 7.5 or for NMR for Par14/PPIase 50 mM KPi buffer pH 6.4–6.8 and Par17 50 mM HEPES, and 150 mM NaCl_2_). If required, proteins were rebuffered three times in the desired buffer using a Centricon 5000 MWCO (Millipore, Merck, Darmstadt, Germany). Protein concentrations were determined by measuring the absorption at 280 nm using the extinction coefficients calculated from the respective amino acid sequences. The protein concentration of the Par17 N‐terminus was determined by applying the Bradford method.

### 5.3 Protein Cross‐Linking

#### 5.3.1 DSSO Cross‐Linking

DSSO (Thermo Scientific, Darmstadt, Germany) was dissolved in DMSO yielding a concentration of 50 mM (stock solution). The proteins (final concentration: Par14, 0.3 mM; Par17, 0.15 mM) were cross‐linked with a 3.3‐fold excess of the DSSO in PBS buffer (pH 7.5). The solution was incubated for 1 h at 25°C and the cross‐linking reaction was stopped with TRIS buffer (final concentration 1 mM, pH 7.5).

#### 5.3.2 Photo Cross‐Linking

For photo‐reactive cross‐linking proteins were expressed by *E. coli* in the presence of photo‐leucine and photo‐methionine (Thermo Scientific, Darmstadt, Germany) and purified. The correct folding was verified by circular dichroism spectroscopy (Figure S4). For cross‐linking a solution of labeled proteins (250 μL of Par14, 0.2 μM, and Par17, 0.07 μM) was irradiated with UV light of 365 nm for 30 min one ice with a distance from the UV source of 1 cm.

#### 5.3.3 Species Separation

Cross‐linked monomeric and dimeric species were separated, by analytical size exclusion chromatography (50 mM HEPES, 150 mM NaCl_2_, pH 7.5).

### 5.4 CD Spectroscopy

For recording of CD‐spectra, proteins were diluted in salt‐free buffer (50 mM KPi buffer, pH 7.5) to a final concentration of 25 μM in 200 μL volume. Measurements were performed on a J‐710 Spectropolarimeter (JASCO GmbH, Pfungstadt, Germany) at 25°C and the spectrum of the buffer was subtracted for baseline correction.

### 5.5 Mass Spectrometry

#### 5.5.1 Sample Clean‐Up for Liquid Chromatography–Mass Spectrometry (LC–MS)

Samples for LC–MS from cross‐linked proteins were digested in‐solution (ISD). For ISD cross‐linked proteins were reduced with DTT (5 mM) in 6 M urea and 50 mM ammonium bicarbonate (ABC) for 30 min at room temperature. Protein reduction was followed by alkylation with iodoacetamide (IAM, 10 mM also in 50 mM ABC, 30 min, room temperature) and quenching of excess IAM with DTT (final concentration DTT 10 mM). Samples were then digested with LysC for 3 h at 37°C. After adjusting the urea concentration to 0.8 M urea the samples were digested for 16 h with trypsin at 37°C. The digestion was stopped by adding formic acid (FA) to a final concentration of 0.5%. The supernatant containing the digestion products was passed through home‐made glass microfiber StageTips (GE Healthcare; poresize: 1.2 μM; thickness: 0.26 mm). Cleared tryptic digests were then desalted on home‐made C18 StageTips as described. Briefly, peptides were passed over a two disc StageTip. After elution from the StageTips, samples were dried using a vacuum concentrator (Eppendorf) and the peptides were taken up in 0.1% FA solution (10 μL). The thus prepared samples were directly used for single play MS/MS experiments (see below for details). For double play MS/MS experiments samples were fractionated on homemade SCX StageTips as described [54]. Briefly, the analytes were immobilized on 0.1% FA pre‐equilibrated SCX StageTips. Peptides were then eluted using 20 μL salt plugs with increasing ABC concentration (0, 10, 50, 100, 150, 200, 250, 300, 400, 500, and 1000 mM ABC). The collected fractions were then dried in a vacuum concentrator, taken up in 10 μL 0.1% FA and directly used for LC–MS analysis.

#### 5.5.2 LC/MS/MS

Experiments were performed on an Orbitrap Elite or Orbitrap Fusion Lumos mass spectrometer (Thermo Fischer Scientific, Waltham, Massachusetts, USA) that were coupled to an EASY‐nLC 1000 or 1200 liquid chromatography (LC) system (Thermo Fisher scientific, Waltham, Massachusetts, USA). The LCs were operated in the one‐column mode. The analytical column was a fused silica capillary (inner diameter 75 μm × 35–45 cm) with an integrated PicoFrit emitter (New Objective, Woburn, USA) packed in‐house with Reprosil‐Pur 120 C18‐AQ 1.9 μm (Dr. Maisch). The analytical column was encased by a column oven (Sonation, Biberach an der Riß, Germany) and attached to a nanospray flex ion source (Thermo Fischer scientific, Waltham, Massachusetts, USA). The column oven temperature was adjusted to 45°C or 50°C during data acquisition. The LC was equipped with two mobile phases: solvent A (0.1% or 0.2% formic acid, FA, in water) and solvent B (0.1% or 0.2% FA, 20% H_2_O, in acetonitrile, ACN). All solvents were of UHPLC (ultra‐high‐performance liquid chromatography) grade (Honeywell, Seelze, Germany). Peptides were directly loaded onto the analytical column with a maximum flow rate that would not exceed the set pressure limit of 980 bar (usually around 0.5–0.8 μL/min). Peptide solutions (SCX fractionated or non‐SCX fractionated) were subsequently separated on the analytical column using different gradients (for details see Table S7).

The mass spectrometers were operated using Xcalibur software (Elite: v2.2 SP1.48; Lumos: v4.3.7.3.11). The mass spectrometers were set in the positive ion mode. Precursor ion scanning (MS1) was performed in the Orbitrap analyzer (FTMS; Fourier Transform Mass Spectrometry) with the internal lock mass option turned on (lock mass was 445.120,025 *m*/*z*, polysiloxane) [55]. MS2 Product ion spectra were recorded only from ions with a charge bigger than +2 and in a data dependent fashion in the FTMS. MS3 product ion spectra were only triggered when the targeted mass difference for DSSO (31.9721 Da) was detected. MS3 spectra were recorded in the IT (ion trap). All relevant MS settings (Resolution, scan range, AGC, ion acquisition time, charge states isolation window, fragmentation type and details, cycle time, number of scans performed, and various other settings) for the individual experiments can be found in Table S7.

#### 5.5.3 Data Analysis

The raw data files for DSSO cross linking experiments were directly processed in Thermo Scientific Proteome Discoverer (PD, version 2.2. or 2.4.) with the add‐on node XlinkX [56]. The MS1 and MS2 spectra were extracted using the “Spectrum Selector” node with the default settings. In the “Scan Event Filter” the activation type (HCD or CID/ETD) and the MS order were adjusted depending on the experiment. In the “XlinkX Detect” node the DSSO cross linker was selected as “Crosslink Modification” (158.004 Da, Specificity STYK). From here the analysis branched off to the “XlinkX search” or a “SEQUEST” search. The following settings were used for the XlinkX search: precursor ion mass tolerance, 10 ppm; product ion mass tolerance, 20 ppm; fixed modification, Cys carbamidomethylation; variable modification, Met oxidation; allowed number of miss‐cleavages, 2. All MS/MS spectra were searched against the sequences of interest concatenated to the Uniprot *E. coli* database (one protein per gene; retrieved in December 2018, containing 4308 target protein entries). FDR calculation (“XlinkX validation” node) is based on a target‐Decoy approach. All peptides not considered to be cross‐linked were analyzed with SEQUEST. The same database as in the XlinkX search was used. The settings are as follows: precursor ion mass tolerance, 10 ppm; fragment ion mass tolerance, 0.1 Da; fixed modification, Cys carbamidomethylation; variable modification, Met oxidation, DSSO (non cross‐linked), DSSO amidated and DSSO hydrolyzed; allowed number of miss‐cleavages was set to 2. The output files from XlinkX were submitted to the xiNET website for visualization.

Experiments where Leu and Met were replaced by photo‐Leu and photo‐Met were analyzed using StavroX (v3.6.6.6) [57]. To this end photo‐Leu and photo‐Met were added as custom amino acids (Name: photo‐Met; OneLetterAbbr: x; MonoisotopicMass: 130.0745; ChemicalFormula: C_6_H_9_N_3_O and Name: photo‐Leu; OneLetterAbbr: #; MonoisotopicMass: 125.0589; ChemicalFormula: C_5_H_7_N_3_O). Both amino acids were setup as cross linkers (Name: ‐N2; Site 1: x or #; site 2: ACDEFGHIKLMNPQRSTVWYx#; mass: −28.006). The Raw files were first converted to mzML format using PD2.2./2.4. The detailed search settings can be found in Table S7. The results from all searches were manually evaluated.

#### 5.5.4 Data Availability

The mass spectrometry proteomics data for the on‐bead digestions have been deposited to the ProteomeXchange Consortium via the PRIDE [58] partner repository (https://www.ebi.ac.uk/pride/archive/) with the dataset identifier PXD053984. During the review process the data can be accessed via a reviewer account (Username: reviewer_pxd053984@ebi.ac.uk; Password: OoH2jhSMqf9X).

### 5.6 Protease‐Coupled Isomerase Assay (PPIase Assay)

We determined the isomerase activity using the PPIase assay described by [30]. The lyophilized peptides were dissolved in an adequate volume of 0.5 mM‐LiCl/TFE buffer to obtain a 15 mM stock solution. To achieve a stable *cis*/*trans* ratio the mixture was incubated overnight at 4°C. The next day a stock solution of 350 μM α‐chymotrypsin in PBS was incubated on ice for at least 20 min. For measurements without PPIase PBS buffer (pH 6.8) was mixed with α‐chymotrypsin to a final concentration of 35 μM in 1 mL. A baseline was recorded for 5 min (Cary 100Bio UV/Vis spectrophotometer, Varian/Agilent, Santa Clara, US, *λ*: 390 nm, bandwidth: 1.0 nm, average time between measurements: 0.5 s, temperature: 10°C). After 5 min the substrate peptide was added to a final concentration of 75 μM in 1 mL [59]. The solution was mixed by inverting the cuvette three times and the reaction was recorded for 15 min. Measurements in the presence of PPIase were performed in the same way, except that before the baseline measurement, the appropriate isomerase was added in a final concentration of 2 μM in 1 mL in addition to PBS and α‐chymotrypsin.

For the data analysis, a mean value of the baseline was subtracted (average of the first 200 data points). The measured values were normalized according to the respective maximum of the measurement. To determine the rate constant, the program graph pad was used, and non‐linear curve fitting was applied for each measurement (exponential, one‐phase association).

(1) $$Y=Y_{0}+\left(\text{Plateau}-Y_{0}\right)∙\left(1-e^{\left(-K∙x\right)}\right)$$

where *x* is the time (min), *Y* is the absorbance, *Y*_0_ is the absorbance when *x* = 0, Plateau is the *Y* value at infinite times, *K* is the rate constant (min^−1^).

The *K*_cat_/*K*_M_ value was calculated according to Equation (2):

(2) $$\frac{k_{\mathrm{cat}}}{K_{M}}=\frac{k_{\mathrm{obs}-}\;k_{\text{therm}}}{\left[\text{PPIase}\right]}$$

where *k*_cat_/*K*_M_ is the catalytic efficiency (M^−1^ s^−1^), *k*_obs_, rate constant of the observed reaction (s^−1^), *k*_therm_ is the rate constant of the thermic reaction (s^−1^), and [PPIase] is the concentration of PPIase used in the assay (M).

The obtained data are fitted to Equation (2) using a pseudo first‐order assumption. This is the case, if *k*_obs_ values behave linearly with rising PPIase concentrations. To prove linearity, the assay was performed with increasing concentrations of Par17 (Figure S5).

### 5.7 NMR Spectroscopy

For NMR measurements, purified and concentrated proteins in salt‐free buffers were mixed with 10% D_2_O and 1 μL DSS standard. Spectra were measured on an ultra shield 700‐NMR (Bruker, Billerica, US) with a triple resonance cryoprobe (700 MHz). For recording and processing of data, the software Topspin 3.5 was used. Analysis of spectra was carried out using Cara analysis software 2.3.1 (Rochus L.J. Keller) and/or CcpNmr Analysis 2.3.1 (Department of Molecular and Cell Biology, University of Leicester, UK).

*Comparison of spectra of full‐length parvulin proteins and PPIase domain*: ^15^N‐HSQC spectra were recorded using the program hsqcetf3gpsi2 from the Bruker standard library. Samples were measured at 25°C under identical buffer conditions (50 mM *K*_Pi_ buffer, pH 6.4). Chemical shift changes were calculated using the following equation:

(3) $$∆\delta =\sqrt{{\left(∆\delta_{H}\right)}^{2}+{\left(∆\delta_{N}∙0.154\right)}^{2}}$$

Δ*δ* is the combined chemical shift, Δ*δ*_H_ is the a chemical shift in the H‐dimension, and Δ*δ*_N_ is the a chemical shift in the N‐dimension.

*Titration of substrate peptides to* ^*15*^ *N‐labeled full‐length Par17 and the N‐terminus of Par17*: The following substrate peptides were diluted in salt‐free 50 mM KPi buffer pH 6.7 to a concentration of 15 mM (Suc: Succinyl, pNA: para‐Nitroanilin):

**Table:** 

Suc‐A‐E‐P‐F‐pNA	Suc‐A‐R‐P‐F‐pNA	Suc‐A‐E‐A‐F‐pNA
Suc‐A‐V‐P‐F‐pNA	Suc‐A‐K‐P‐F‐pNA	Suc‐A‐E‐P‐F
Suc‐A‐G‐P‐F‐pNA		

They were added stepwise to 200 μL of the labeled parvulins (300 μM in 50 mM KPi buffer pH 6.7). Spectra were recorded with the pulse program Best‐TROSY at 20°C. Combined chemical shifts were calculated as described. Microscopic dissociation constant *K*_D_ was fitted with the following equation (assuming a 1:1 binding model):

(4) $$∆\delta =∆\delta \max\cdot \frac{\left(P+x+K_{D}\right)-\sqrt{{\left(P+x+K_{D}\right)}^{2}-\left(4\cdot x\cdot P\right)}}{2\cdot P}$$

where ∆δmax is the amplitude, *P* is the protein concentration, *X* is the concentration of the peptide.

Differences in chemical shift changes could be measured up to 0.001 ppm (error in peak picking). Changes were interpreted as significant if Δ > 0.004 ppm and when appearing consecutively in a linear sequence element.

The microscopic *K*_D_ describes the binding affinity of the ligand to a single side of an amino acid. This does not necessarily correspond to the macroscopic binding constant obtained by light spectroscopic techniques or ITC.

### 5.8 Atto594 Labeling and Peptide Spotter Array

β‐Actin peptide libraries were produced by automatic SPOT synthesis by Fmoc (9‐fluorenylmethyloxycarbonyl) chemistry on Whatmancellulose 50 membranes (Peptide Synthesizer, MultiPep RSi, INTAVIS Bioanalytical Instruments AG, Koeln, Germany). The dried membranes were activated in 100% ethanol for 10 min, washed with PBS, and incubated with blocking buffer (5% BSA in PBS) for 1 h at room temperature. Next, they were incubated overnight with Atto594‐labeled Par14/Par17 and free dye for control. Par17 and Par14 were labeled with Atto594 NHS‐ester according to the company's instructions (ATTO‐TEC GmbH, Siegen, Germany). Membranes were washed with PBS and dried at 37°C. Followed by the detection of the bound proteins (Typhoon FLA 9000 Imaginer *λ*_excitation_ = 523 nm, *λ*_emission_ = 665 nm).

### 5.9 SDS‐Page

Samples were supplemented with 5x SDS sample buffer and boiled at 95°C for 5 min. Samples were loaded onto a self‐cast SDS‐Gel. The proteins in the gels were stained with coomassie blue.

### 5.10 Model Calculation With YASARA

#### 5.10.1 Model of Par17

A model for Par17 was calculated on the basis of the sequence of Par17 and the structure of the PPIase domain (PDB: 3UI6), [33] using the software I‐TASSER [60]. Molecular dynamics simulations of the model generated by I‐TASSER were performed with the help of the YASARA Structure Suite (v11.12.31) and a YASARA2 force field using the experimental cross‐links between the PPIase domain and the N‐terminus as distance restraints. Additionally, all amino acids within the PPIase domain that did not shift when the Nterminus of Par17—was present were fixed, except the amino acids that were part of a cross‐link. For the simulation, the cubic simulation cell size was 78.5 Å per edge. The simulation was performed with an explicit water model, periodic boundaries, and drift correction. The temperature was set at 298 K and the simulation was run with 0.9% (m/m) NaCl, pH 7.4 with 2.5 fs step width and 7.8 Å cutoff for Van der Waals and real‐space Coulomb forces.

### 5.11 Docking With Haddock

#### 5.11.1 Model of the Par17 and Actin Complex

The interaction of the Par17‐actin complex was modeled in HADDOCK [39] using a cryo‐EM structure of one actin domain (PDB‐ID: 6ANU) and the ITASSER‐generated Par17 model‐ based on the calculated PPIase domain structure. The active residuals for Par17 were defined by the NMR experiments. All amino acids that showed chemical shift changes after actin addition were included and set as ambiguous interaction restraints. For actin, active residues were defined as amino acids bound by Par17 in the spotter array that are exposed on the surface of actin. The inter‐domain crosslinks obtained by DSSO cross‐linking were used as unambiguous restraints for docking. Default HADDOCK scaling for energy terms was applied. Rigid body docking resulted in 1000 complex structures, and the best 200 lowest energy structures were selected for semi‐flexible and water refinement. The scoring function yielded a single clear ensemble with a HADDOCK score of 652.7 ± 8.2. A molecular dynamic simulation of the Par17‐actin complex was subsequently performed with YASARA (Elmar Krieger [61] [62]. The settings from the YASARA section were used and the inter‐domain DSSO cross‐links between actin and Par17 were used as distance restraints.

## Author Contributions

**Anna Sternberg:** investigation, writing – original draft, methodology, visualization, writing – review and editing, validation, software. **Jennifer Lynne Borger:** investigation. **Mathilda Thies:** investigation. **Anja Matena:** writing – original draft, writing – review and editing, supervision, visualization, validation, software. **Mike Blueggel:** investigation. **Bianca E. Kamba:** investigation. **Christine Beuck:** investigation. **Farnusch Kaschani:** investigation, writing – original draft. **Markus Kaiser:** supervision. **Peter Bayer:** supervision, writing – original draft, writing – review and editing, conceptualization, validation, formal analysis.

## Supporting information


**Data S1.** Supporting Information.

**Table S2.** Chemical shifts measured between Par14 and Par17.

**Table S3.** Combined chemical shift perturbation of full length Par17 after titration of different. Xaa‐Pro‐peptides for selected amino acids and *K*_D_‐values.

**Table S4.** Photo cross‐links of Par14 extracted from the raw data with a score ≥ 100.

**Table S5.** DSSO crosslinking data of Par17 and Actin exported from program Proteome Discoverer.

**Table S6.** 12‐mers of β‐actin used in the peptide array, identified as possible target regions for Par14 (red), Par17 (blue), and both Par14/17 (purple).

**Table S7.** Intramolecular cross‐linking of hPar14/17 with genetically coded photo amino acids.

**Table S8.** Intermolecular hydrogen bonds within the Haddock hPar17‐Actin complex calculated by YASARA.

**Table S9.** Residues found to be mutated in β‐actin.

**Table S10.** HADDOCK statistics for the most reliable cluster (cluster 1).

**Table S11.** YASARA Console.

## Data Availability

The mass spectrometry proteomics data for the on‐bead digestions have been deposited to the ProteomeXchange Consortium via the PRIDE [58] Partner repository (https://www.ebi.ac.uk/pride/archive/) with the dataset identifier PXD053984. During the review process the data can be accessed via a reviewer account (Username: reviewr_pxd053984@ebi.ac.uk; Password: OoH2jhSMqf9X).
