# Supplementary material for: The Actin‐Binding Prolyl‐Isomerase Par17 Sustains Its Substrate Selectivity by Interdomain Allostery
Source: Proteins. 2025 Mar 12;93(9):1481–97. doi: 10.1002/prot.26807 (PMC12314576; doi:10.1002/prot.26807)
Supplement: Supplementary file 1 — Data S1. Supporting Information. [file PROT-93-1481-s010.docx]

Suppl. Table ST1 and Suppl. Figures S1-S7

**Supplementary Table ST1:** Mean rate constant k and the k_cat_/K_m_ value of the target proteins for various model substrate peptides Suc-Ala-Xaa-Pro-Phe-pNa calculated from the protease-coupled isomerase assay (measurements were repeated n times). Catalytic activity towards pSer-Pro peptides was not detectable for Par17 in contrast to findings by Monti and coworkers (Monti et al. 2022).

| Xaa | k = k_observed_ - k_thermic_ [min^-1^] and number of measurements (n) | | | | | | k_cat_/K_M_ [M^-1^·s^-1^] · 10^3^ | | |
| --- | --- | --- | --- | --- | --- | --- | --- | --- | --- |
|  | **Par17** | **n** | **Par14**  **(Par17_Δ1-25_)** | **n** | **PPIase domain (Par17_Δ1-60_)** | **n** | **Par17** | **Par14**  **(Par17_Δ1-25_)** | **PPIase domain**  **(Par17_Δ1-60_)** |
| Ala | 0.017 ± 0.043 | 10 | 0.770 ± 0.608 | 9 | 0.508 ± 0.262 | 9 | 0.1 | 6.4 | 4.2 |
| Arg | 0.470 ± 0.037 | 3 | 0.582 ± 0.094 | 3 | 0.683 ± 0.064 | 3 | 3.9 | 4.9 | 5.7 |
| Asn | <0.01 ± 0.157 | 6 | 0.192 ± 0.322 | 8 | 0.173 ± 0.303 | 6 | <0.1 | 1.6 | 1.4 |
| Asp | 0.013 ± 0.013 | 6 | 0.028 ±0.063 | 6 | 0.015 ± 0.032 | 6 | 0.1 | 0.2 | 0.1 |
| Gln | 0.040 ± 0.022 | 6 | 0.537 ± 0.424 | 11 | 0.618 ± 0.367 | 9 | 0.3 | 4.4 | 5.2 |
| Glu | <0.01 ± 0.029 | 6 | 0.267 ± 0.011 | 3 | 0.114 ± 0.074 | 8 | <0.1 | 2.2 | 1.0 |
| Gly | <0.01 ± 0.029 | 5 | 0.390 ± 0.457 | 6 | 0.275 ± 0.214 | 5 | 0.3 | 3.3 | 2.3 |
| His | 0.036 ± 0.006 | 3 | 0.067 ± 0.009 | 3 | 0.068 ± 0.029 | 3 | 0.3 | 0.6 | 0.6 |
| Ile | 0.013 ± 0.013 | 6 | 0.146 ± 0.270 | 5 | 0.147 ± 0.019 | 6 | 0.1 | 1.2 | 1.2 |
| Leu | 0.063 ± 0.021 | 7 | 0.304 ± 0.291 | 10 | 0.223 ± 0.178 | 10 | 0.5 | 2.5 | 1.9 |
| Lys | 0.33 ± 0.012 | 3 | 0.412 ± 0.014 | 3 | 0.442 ± 0.006 | 3 | 2.7 | 3.4 | 3.7 |
| Phe | 0.01 ± 0.005 | 4 | 0.268 ± 0.284 | 6 | 0.298 ± 0.197 | 6 | 0.1 | 2.2 | 2.5 |
| Ser | <0.01 ± 0.098 | 6 | 0.332 ± 0.054 | 3 | 0.327 ± 0.273 | 6 | <0.1 | 2.7 | 2.7 |
| Thr | <0.01 ± 0.048 | 3 | 0.229 ± 0.334 | 5 | 0.141 ± 0.218 | 5 | <0.1 | 1.9 | 1.2 |
| Trp | 0.012 ± 0.001 | 3 | 0.148 ± 0.007 | 6 | 0.063 ± 0.034 | 6 | 0.1 | 1.2 | 0.5 |
| Tyr | 0.012 ± 0.005 | 4 | 0.371 ± 0.176 | 6 | 0.198 ± 0.148 | 6 | 0.1 | 3.1 | 1.7 |
| Val | 0.011 ± 0.005 | 6 | 0.408 ± 0.432 | 6 | 0.289 ± 0.166 | 5 | 0.1 | 3.4 | 2.4 |


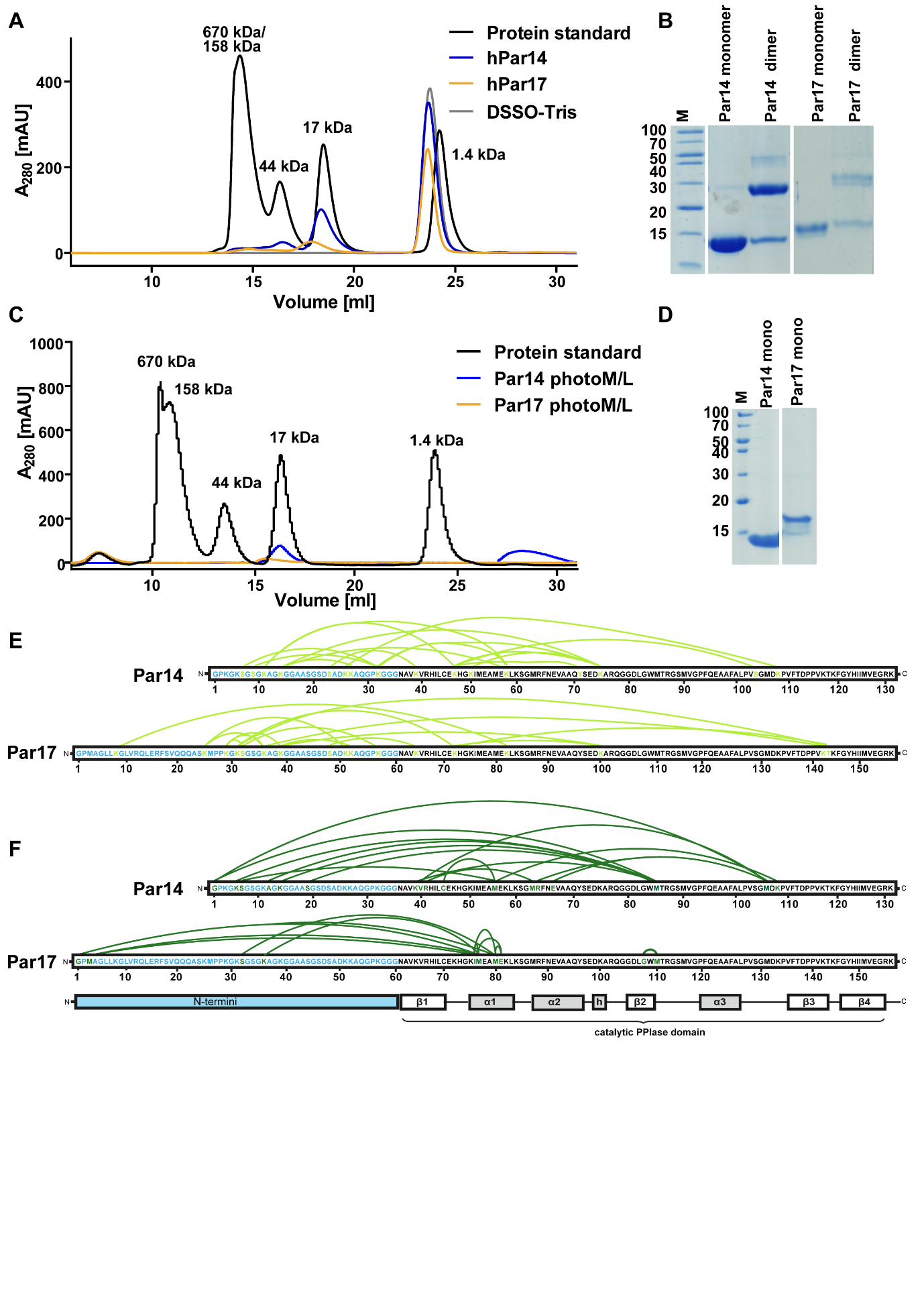


Supplementary Figure S1: Intramolecular cross-linking of Par14 and Par17. Analytical gel-filtration chromatogram measured at 280 nm of cross-linked Par17 or Par14 by **A:** DSSO or **C:** Photo-reactive amino acids. The peaks of the protein standard (black curve) are labeled with the corresponding protein size. SDS gels of the fractions from the peaks in the gel permeation chromatography of **B:** DSSO cross-linking and **D:** Photo cross-linking experiment. Intramolecular cross-links identified by mass spectrometry by **E:** DSSO cross-linking with a score ≥ 90 or **F:** Photo cross-linking with a score ≥ 100. The N-termini of Par14 or Par17 are highlighted in light blue.


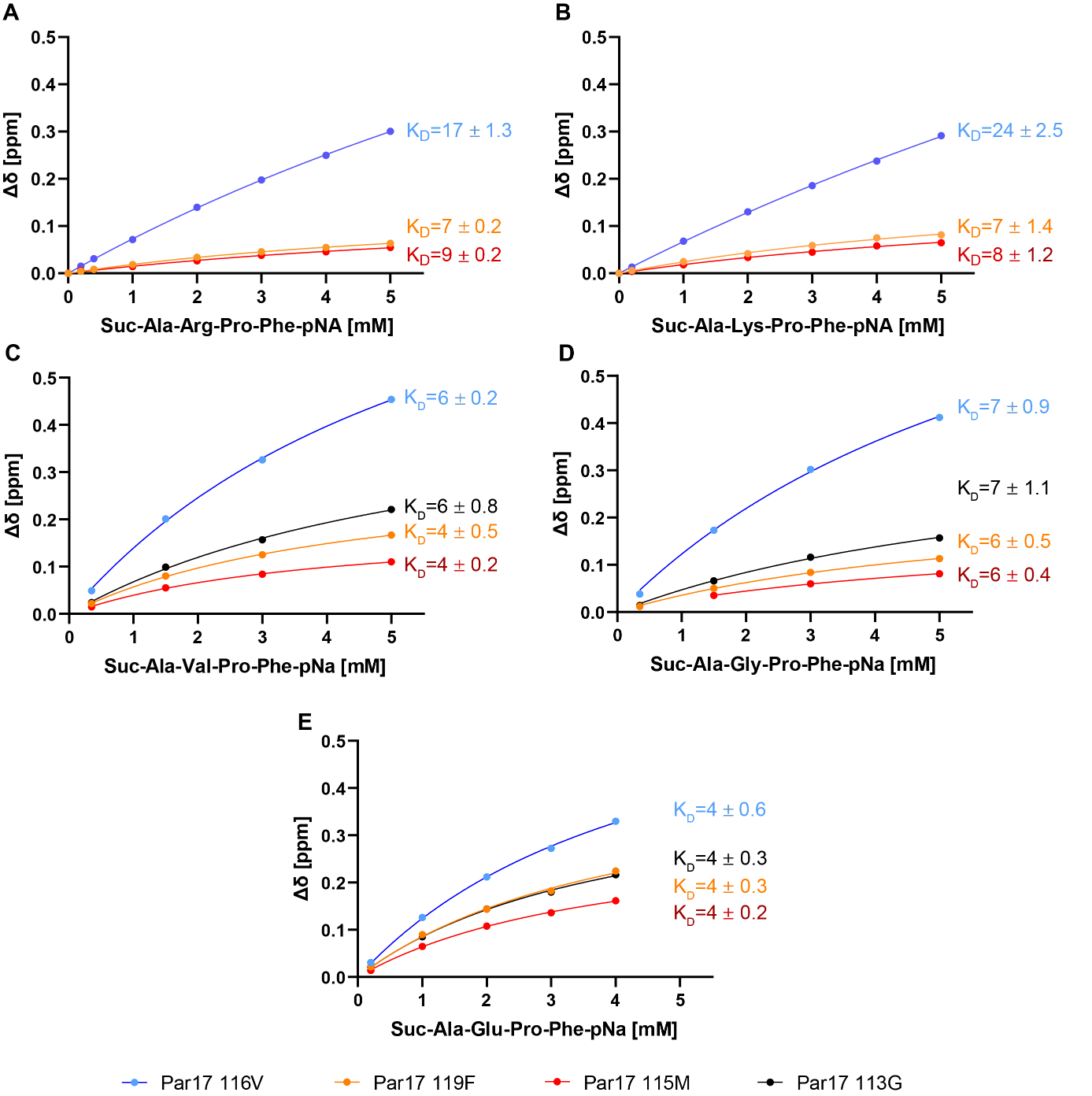


Supplementary Figure S2**: NMR titration of Par17 with model substrate peptides using ^15^N-HSQC spectra.** Chemical shift changes of resonances of residues of Par17 are plotted against the concentration of model substrate in which either arginine (**A**), lysine (**B**), valine (**C**), glycine (**D**), or glutamate (**E**) is preceding proline. The dissociation constant K_D_ [mM] was fitted using equation 4 and is plotted in the color of the corresponding amino acid. R^2^ = [0.995; 0.999] for all fittings.


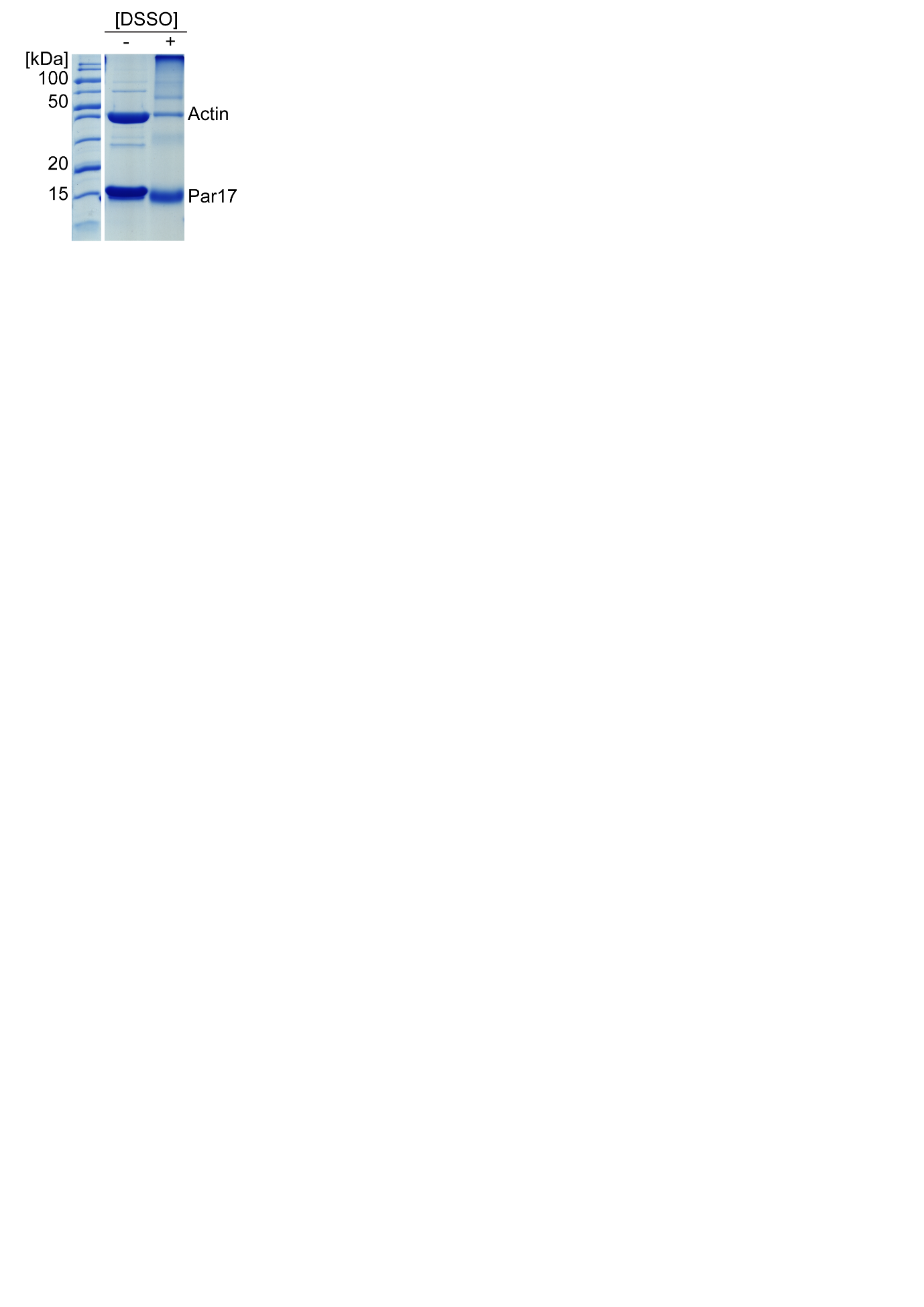


Supplementary Figure S3**: SDS-gel of DSSO Cross-linking of Par17 with actin.** For DSSO cross-linking of actin (Cytoskeleton Inc., Cat.#APHL99) and Par17 both were mixed (10 μM and 25 μM respectively in a total volume of 140 μM; 50 mM HEPES, 150 mM NaCl2, pH 7.5) and incubated for 30 min at 4 °C. Then a sample was taken (DSSO-) for SDS-PAGE. DSSO was added in a 3.3-fold excess (0.8 mM) and the reaction was stopped after 10 min with TRIS buffer. A sample was taken (DSSO+) for SDS-PAGE. Samples were applied to an SDS gel in addition to a marker (ThermoFisher, PageRuler, broad range, unstained). The bands of native proteins and the protein size of the marker were labeled.

**
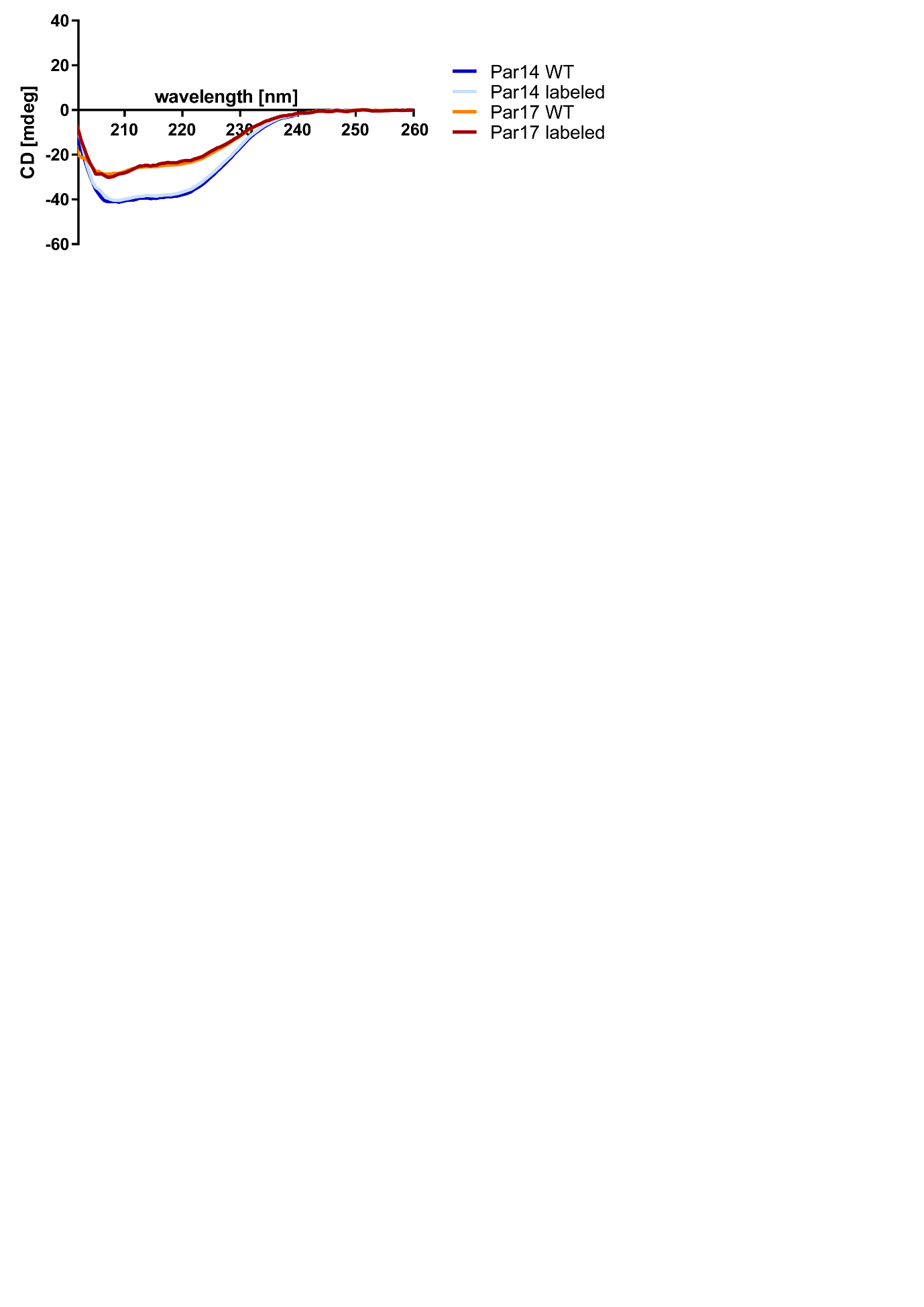
**

Supplementary Figure S4: CD spectra of WT parvulins compared to photo-reactive amino acid labeled parvulins. The proteins were measured at 25 °C in their native buffer (50 mM HEPES, 150 mM NaCl) diluted with 50 mM KPi buffer to a concentration of 25 µM. CD spectra were measured from 190 to 260 nm. The wavelengths between 190 nm and 202 nm were omitted because the salt in the buffer influenced the measured values in this region. Each spectrum was recorded with 25 scans. For comparison, the intensity of Par14 WT was multiplied by a factor of 1.19 and that of Par17 was labeled by 1.5. The shape of the curves of the labeled proteins and the native proteins were quite similar.


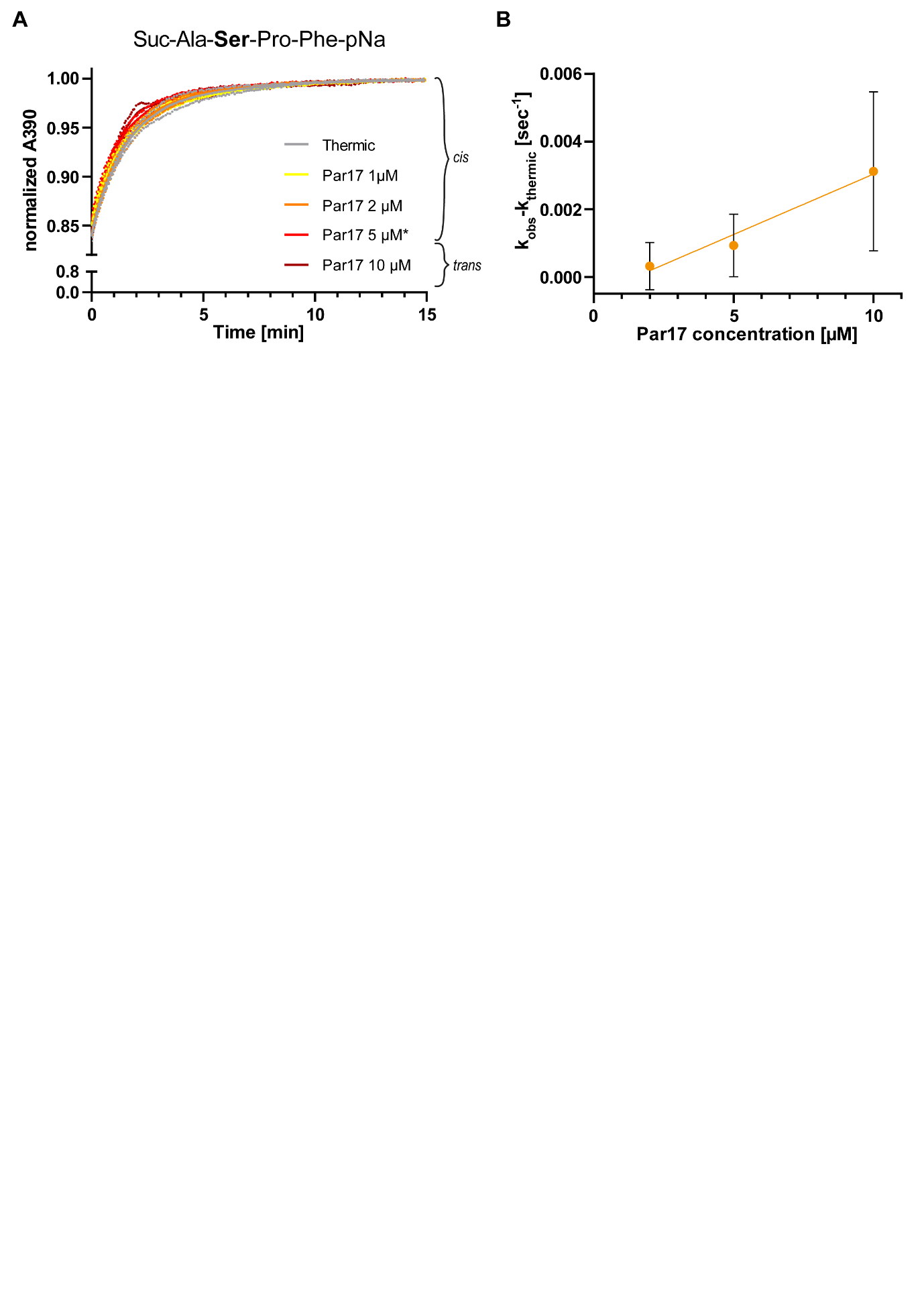


**Supplementary Figure S5: Linearity of the rate constant of the catalyzed reaction with the amount of added enzyme Par17.** A: The mean change in absorbance at 390 nm after the addition of the model substrate Suc‑Ala‑Ser-Pro-Phe-pNa in the presence and absence of different concentrations of Par17 are depicted. Three replicates each were measured (asterisk marked: only two replicates). Dots represent the standard deviation of the corresponding mean. On the right side of the graph, the portions of *cis* and *trans* isomers at the beginning of the measurement are displayed. B: The calculated rate constant from measurements with different enzyme concentrations of Par17 for the substrate Suc-Ala-Ser-Pro-Phe-pNa are plotted against the concentration. A linear fit for the data points is shown.


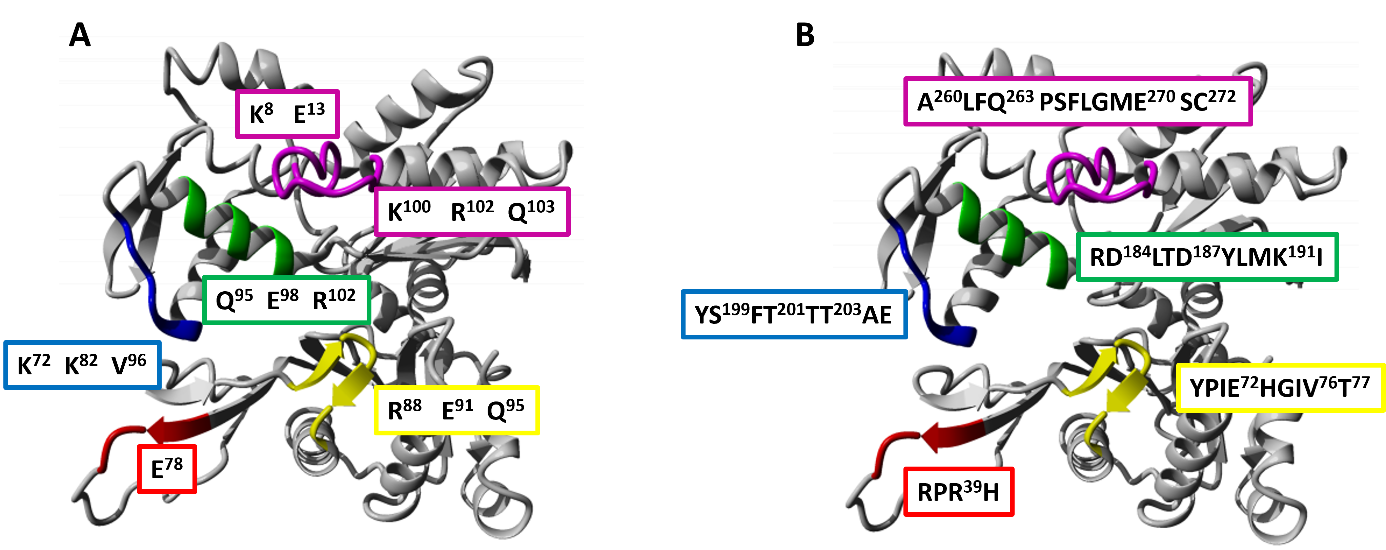


**Supplementary Figure S6:** **Structural model of β-actin (6ANU, gray).** The hPar17-interface as determined by intermolecular hydrogen bonding is coloured (red: D-loop aa 37-41; yellow: Sensor loop aa 70-78; green: helix 5 aa 183-192; blue: Threonine-rich region aa 198-203; magenta: hydrophobic plug aa 260-272). Sequences of β-actin forming hydrogen bonds to hPar17 are framed in the above colours (right). Residues from hPar17 forming hydrogen bonds to a relevant region of actin are framed in the corresponding colour (left). Hydrogen bonded residues are denoted by their sequence position.


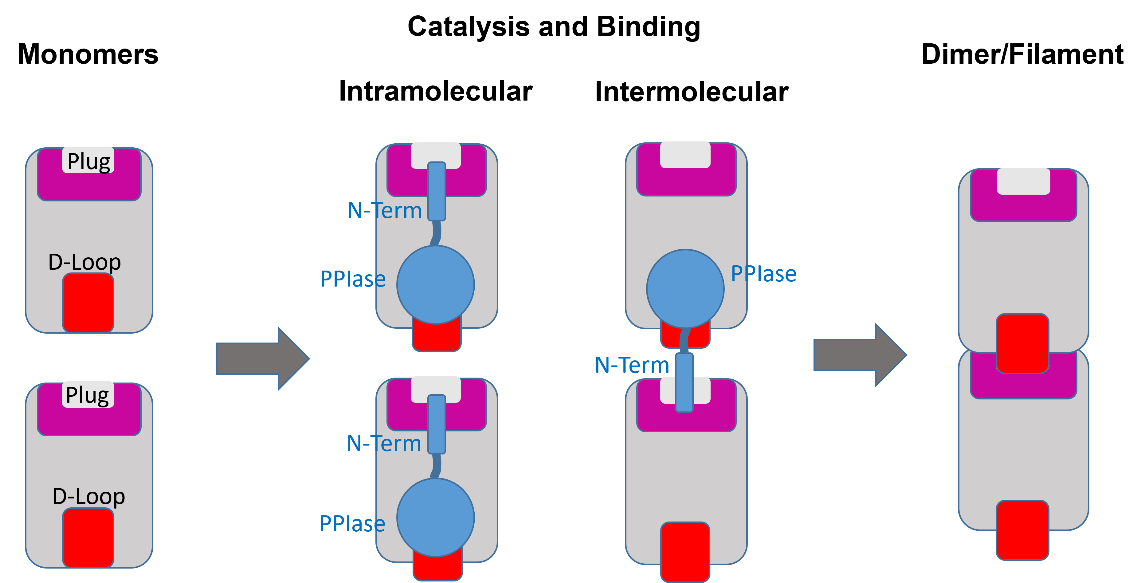


**Supplementary Figure S7: Hypothetical model of the Par17-β-actin binding and catalysis.** Blue: Par17; gray: β-actin; red: D-loop; magenta: hydrophobic plug.
