# Supplementary material for: The Actin‐Binding Prolyl‐Isomerase Par17 Sustains Its Substrate Selectivity by Interdomain Allostery
Source: Proteins. 2025 Mar 12;93(9):1481–97. doi: 10.1002/prot.26807 (PMC12314576; doi:10.1002/prot.26807)
Supplement: Supplementary file 2 — Table S2. Chemical shifts measured between Par14 and Par17. [file PROT-93-1481-s007.pdf]

Chemical shifts measured between Par14 and Par17

Shift 1A	Shift 1B	Δ 1 (ppm)	Shift 2A	Shift 2B	Δ 2 (ppm)	Shift Dist	Seq Num	Amino Acids
8,35435	8,41962	0,06527	121,74911	121,95942	0,21031	0,07286	29	K
8,2322	8,24376	0,01155	121,10443	121,10219	-0,00224	0,01156	31	K
8,46121	8,47399	0,01278	110,21493	110,20296	-0,01197	0,01291	40	G
8,21008	8,21823	0,00815	108,85443	108,89242	0,03799	0,01003	41	G
8,21779	8,22967	0,01189	115,20184	115,20813	0,00628	0,01193	44	S
8,18854	8,20121	0,01266	115,66975	115,65074	-0,01901	0,013	46	S
8,10983	8,13264	0,02281	121,63083	121,72948	0,09865	0,02741	52	K
8,36346	8,37068	0,00723	110,25362	110,18646	-0,06716	0,01262	58	G
9,16552	9,16857	0,00304	125,09218	125,22693	0,13475	0,02097	73	H
8,89858	8,89844	-1,41E-04	104,37768	104,30908	-0,0686	0,01056	74	G
7,38347	7,39342	0,00995	118,61162	118,67308	0,06146	0,01374	76	I
7,35525	7,3719	0,01665	119,08708	119,10559	0,01852	0,01689	77	M
8,87994	8,9138	0,03386	121,44122	121,48466	0,04344	0,03451	100	K
8,62171	8,63772	0,01601	106,09598	106,11803	0,02205	0,01637	105	G
10,2214	10,23524	0,01385	129,56531	129,60114	0,03583	0,0149	109	W
8,38687	8,40334	0,01647	116,93668	116,91798	-0,0187	0,01672	116	V
8,77579	8,79119	0,0154	111,66916	111,68159	0,01243	0,01552	117	G
7,31377	7,29955	-0,01421	116,91008	116,8874	-0,02268	0,01464	119	F
8,27054	8,284	0,01346	118,54719	118,56609	0,01889	0,01377	120	Q
8,36492	8,37516	0,01024	118,5602	118,57506	0,01486	0,01049	123	A
8,72538	8,73349	0,00812	117,70847	117,66764	-0,04083	0,01027	144	K
8,57895	8,58986	0,01091	118,13331	118,16132	0,02801	0,01173	145	F

Amino acids are located within the catalytic domain
