# Supplementary material for: The Actin‐Binding Prolyl‐Isomerase Par17 Sustains Its Substrate Selectivity by Interdomain Allostery
Source: Proteins. 2025 Mar 12;93(9):1481–97. doi: 10.1002/prot.26807 (PMC12314576; doi:10.1002/prot.26807)
Supplement: Supplementary file 3 — Table S3. Combined chemical shift perturbation of full length Par17 after titration of different. Xaa‐Pro‐peptides for selected amino acids and K D‐values. [file PROT-93-1481-s011.pdf]

Combined chemical shift perturbation of full length Par17 after titration of different Xaa-Pro-peptides for selected amino acids and KD-values

Xaa-Lys peptide [mM]	116V	119F	115M	
0	0	0	0	[ppm]
0,2	0,01300091	0,00604921	0,00414271	
1	0,06817833	0,02459781	0,01818939	
2	0,13037308	0,04192915	0,03362789	
3	0,18551846	0,05880948	0,04495973	
4	0,23776807	0,07526515	0,05828389	
5	0,29129267	0,08126177	0,06478417	
KD	24	7	8	mM

Xaa-Arg peptide [mM]	116V	119F	115M	
0	0	0	0	[ppm]
0,2	0,01300091	0,00418543	0,0060079	
0,4	0,06817833	0,00841748	0,00801333	
1	0,13037308	0,01835824	0,0143348	
2	0,18551846	0,03376697	0,02655282	
3	0,23776807	0,04577626	0,03809928	
4	0,29129267	0,05474353	0,0454705	
5	24	0,06351885	0,0546769	
KD	17	7	9	mM

Xaa-Glu peptide [mM]	116V	119F	115M	113G	
0,2	0,03056	0,02147	0,01409	0,02117	[ppm]
1	0,12599	0,0896	0,06465	0,08535	
2	0,21191	0,14351	0,10754	0,14414	
3	0,27245	0,18213	0,13595	0,17998	
4	0,32999	0,22445	0,16135	0,21636	
KD	4	4	4	4	mM

Xaa-Val peptide [mM]	116V	119F	115M	113G	
0,35	0,049	0,022	0,015	0,024	[ppm]
1,5	0,201	0,08	0,055	0,099	
3	0,326	0,125	0,084	0,157	
5	0,454	0,167	0,11	0,221	
KD	6	4	4	6	mM

Xaa-Gly peptide [mM]	116V	119F	115M	113G	
0,35	0,038	0,012		0,014	[ppm]
1,5	0,173	0,05	0,035	0,066	
3	0,302	0,084	0,06	0,116	
5	0,412	0,113	0,081	0,157	
KD	7	6	6	7	mM
