# Supplementary material for: The Actin‐Binding Prolyl‐Isomerase Par17 Sustains Its Substrate Selectivity by Interdomain Allostery
Source: Proteins. 2025 Mar 12;93(9):1481–97. doi: 10.1002/prot.26807 (PMC12314576; doi:10.1002/prot.26807)
Supplement: Supplementary file 4 — Table S4. Photo cross‐links of Par14 extracted from the raw data with a score ≥ 100. [file PROT-93-1481-s009.pdf]

Photo cross-links of Par14 extracted from the raw data with a score ≥ 100

Number	Score	Pos1	Amino acid	Pos2	Amino Acid	shown in Fig S1,	shown in Fig S1, F	Distance
1	202	45	C	55	M	yes		
2	200	1	G	85	M	yes	yes	23
3	197	1	G	85	M	double		
4	174	6	K	85	M	yes	yes	17,5
5	172	14	K	85	M	yes	yes	19,1
6	166	45	C	55	M	double		
7	156	14	K	85	M	double		
8	153	39	K	85	M	yes		
9	145	6	K	85	M	double		
10	139	1	G	85	M	double		
11	127	12	A	85	M	yes	yes	24,5
12	121	1	G	106	M	yes	yes	42
13	116	7	S	55	M	yes	yes	46,4
14	116	1	G	85	M	double		
15	115	1	G	106	M	double		
16	114	41	R	62	M	yes		
17	110	1	G	106	M	double		
18	108	40	V	106	M	yes		
19	107	19	S	85	M	yes	yes	21,9
20	106	66	E	106	M	yes		
21	102	108	K	55	M	yes		
22	102	64	R	106	M			
23	100	1	G	106	M			

Photo cross-links of Par17 extracted from the raw data with a score ≥ 100

Score	Peptide 1	Protein 1	From	To	best linkage position peptide 1			Peptide2	Protein 2	From	To	best linkage position peptide 2			shown in Fig S1, F	shown in Fig S1, F	Distance
204	{GPMAGLLK]	>hPar17	0	8	{0	1	G	[IxEAMEK]	>hPar17	76	82	x2	77	M	yes	yes	33,4
200	{GPMAGLLK]	>hPar17	0	8	{0	1	G	[IxEAMEK]	>hPar17	76	82	x2	77	M	double		
197	{GPMAGLLK]	>hPar17	0	8	{0	1	G	[IxEAMEK]	>hPar17	76	82	x2	77	M	double		
195	{GPxAGLLK]	>hPar17	0	8	x3	3	M	[IMEAMEK]	>hPar17	76	82	M2	77	M	yes	yes	43,9
191	{GPxAGLLK]	>hPar17	0	8	x3	3	M	[IMEAMEK]	>hPar17	76	82	M2	77	M	double		
185	{GPxAGLLK]	>hPar17	0	8	x3	3	M	[IMEAMEK]	>hPar17	76	82	M2	77	M	double		
167	{GPMAGLLK]	>hPar17	0	8	{0	1	G	[IMEAxEK]	>hPar17	76	82	x5	80	M	yes	yes	27,9
162	{GPMAGLLK]	>hPar17	0	8	{0	1	G	[IMEAxEK]	>hPar17	76	82	x5	80	M	double		
161	[QGGD#GWxTR]	>hPar17	103	112	G6	108	G	[QGGD#GWxTR]	>hPar17	103	112	x8	110	M	yes		
147	{GPMAGLLK]	>hPar17	0	8	{0	1	G	[IMEAxEK]	>hPar17	76	82	x5	80	M	double		
139	[IMEAMEK]	>hPar17	76	82	E6	81	E	[IxEAMEK]	>hPar17	76	82	x2	77	M	yes		
131	{GPMAGLLK]	>hPar17	0	8	{0	1	G	[IxEAMEK]	>hPar17	76	82	x2	77	M	double		
126	{GPxAGLLK]	>hPar17	0	8	x3	3	M	[IMEAMEK]	>hPar17	76	82	I1	76	I	yes	yes	32,8
121	[ARQGGD#GWxTR]	>hPar17	101	112	G8	108	G	[ARQGGD#GWxTR]	>hPar17	101	112	x10	110	M	double		
116	[ARQGGD#GWxTR]	>hPar17	101	112	G8	108	G	[ARQGGD#GWxTR]	>hPar17	101	112	x10	110	M	double		
115	[IMEAMEK]	>hPar17	76	82	E6	81	E	[IMEAxEK]	>hPar17	76	82	x5	80	M	yes		
114	[QGGD#GWxTR]	>hPar17	103	112	G6	108	G	[QGGD#GWxTR]	>hPar17	103	112	x8	110	M	double		
111	[IMEAMEK]	>hPar17	76	82	I1	76	I	[IxEAMEK]	>hPar17	76	82	x2	77	M	yes		
108	{GPMAGLLK]	>hPar17	0	8	{0	1	G	[IMEAxEK]	>hPar17	76	82	x5	80	M	double		
105	[IMEAMEK]	>hPar17	76	82	I1	76	I	[IMEAxEK]	>hPar17	76	82	x5	80	M	yes		
104	[SGSGKAGK]	>hPar17	32	39	S1	32	S	[HGKIxEAMEK]	>hPar17	73	82	x5	80	M	yes	yes	46,8
103	{GPMAGLLK]	>hPar17	0	8	{0	1	G	[IxEAMEK]	>hPar17	76	82	x2	77	M	double		
103	[ARQGGD#GWxTR]	>hPar17	101	112	G8	108	G	[ARQGGD#GWxTR]	>hPar17	101	112	x10	110	M	double		
102	{GPMAGLLK]	>hPar17	0	8	{0	1	G	[IxEAMEK]	>hPar17	76	82	x2	77	M	double		
102	[ARQGGD#GWxTR]	>hPar17	101	112	G8	108	G	[ARQGGD#GWxTR]	>hPar17	101	112	x10	110	M	double		
101	[SGSGKAGK]	>hPar17	32	39	K5	36	K	[IxEAMEK]	>hPar17	76	82	x2	77	M	yes	yes	53,8
100	[ARQGGD#GWxTR]	>hPar17	101	112	G8	109	G	[ARQGGD#GWxTR]	>hPar17	101	112	x10	110	M	double		.
100	[ARQGGD#GWxTR]	>hPar17	101	112	G8	109	G	[ARQGGD#GWxTR]	>hPar17	101	112	x10	110	M	double		

DSSO cross-links of Par14 extracted from the raw data with a score ≥ 90

Number	Score	Pos1	Par17 Pos		Pos2	Par17 Pos		shown fig S1, E	shown fig S1, E	distance A
1	166,61	14	39	K	75	100	K	yes	yes	30,4
2	157,99	9	34	S	32	57	K	yes	yes	29,3
3	152,39	75	100	K	50	75	K	yes		
4	144,28	108	133	K	26	51	K	yes	yes	41,8
5	130,04	75	100	K	47	72	K	yes		
6	120,87	7	32	S	32	57	K	yes	yes	33,4
7	103,41	14	39	K	57	82	K	yes	yes	45,3
8	98,03	23	48	S	39	64	K	yes	yes	32,7
9	96,84	47	72	K	11	36	K	yes	yes	49
10	95,54	14	39	K	27	52	K	yes		
11	94,68	57	82	K	11	36	K	yes	yes	55,5
12	92,32	104	129	S	47	72	K			
13	92,19	71	96	Y	47	72	K			

DSSO cross-links of Par17 extracted from the raw data with a score ≥ 90

Number	Score	Protein1	Pos1		Pos2		shown in Fig S1, E	shown in Fig S1, E	distance A
1	175,65	hPar17-WT	100	K	75	K	yes		
2	167,23	hPar17-WT	25	K	36	K	yes		
3	164,89	hPar17-WT	36	K	29	K	yes		
4	158,36	hPar17-WT	39	K	100	K	yes	yes	33,3
5	155,51	hPar17-WT	36	K	144	K	yes	yes	30,4
6	147,35	hPar17-WT	32	S	57	K	yes		
7	146,94	hPar17-WT	39	K	29	K	yes		
8	120,65	hPar17-WT	39	K	52	K	yes		
9	119,22	hPar17-WT	8	K	82	K	yes	yes	24,3
10	113,1	hPar17-WT	34	S	29	K	yes		
11	107,32	hPar17-WT	51	K	25	K	yes		
12	99,28	hPar17-WT	143	T	72	K	yes		
13	95,38	hPar17-WT	48	S	64	K	yes	yes	30,1
14	91,91	hPar17-WT	72	K	31	K	yes	yes	32,8
15	91,78	hPar17-WT	143	T	31	K	yes	yes	25
