# Supplementary material for: The Actin‐Binding Prolyl‐Isomerase Par17 Sustains Its Substrate Selectivity by Interdomain Allostery
Source: Proteins. 2025 Mar 12;93(9):1481–97. doi: 10.1002/prot.26807 (PMC12314576; doi:10.1002/prot.26807)
Supplement: Supplementary file 5 — Table S5. DSSO crosslinking data of Par17 and Actin exported from program Proteome Discoverer. [file PROT-93-1481-s004.pdf]

DSSO crosslinking data of Par17 and Actin exported from program Proteome Discoverer

Id	Protein1	PepPos1	PepSeq1	LinkPos1	Protein2	PepPos2	PepSeq2	LinkPos2
1	Par17	37	AGKGGAASGSDSADKK	15	Par17	52	KAQGPKGGGNAVK	6
2	Par17	32	SGSGKAGK	5	Par17	26	MPPKGK	4
3	Par17	37	AGKGGAASGSDSADKK	12	Par17	53	AQGPKGGGNAVK	5
4	Par17	53	AQGPKGGGNAVK	5	Par17	19	VQQQASKMPPK	7
5	Par17	19	VQQQASKMPPK	7	Par17		VRHILCEKHGK	8
6	Par17	89	FNEVAAQYSEDKAR	12	Par17	65	VRHILCEKHGK	8
7	Par17	89	FNEVAAQYSEDKAR	8	P76514	168	FNDLSELRK	5
8	Par17	89	FNEVAAQYSEDKAR	12	Par17	58	GGGNAVKVR	7
9	Par17	89	FNEVAAQYSEDKAR	12	Par17	19	VQQQASKMPPK	7
10	Par17	65	VRHILCEKHGK	8	Par17	76	IMEAMEKLK	7
11	P63261	313	MQKEITALAPSTMK	3	P46850	112	TTGRCKRDK	2
12	Par17	89	FNEVAAQYSEDKAR	12	Par17	73	HGKIMEAMEK	3
13	Par17	19	VQQQASKMPPK	6	Par17	76	IMEAMEKLK	7
14	Par17	19	VQQQASKMPPK	7	Par17	76	IMEAMEKLK	7
15	P63261	51	DSYVGDEAQSKR	11	Par17	76	IMEAMEKLK	7
16	P63261	51	DSYVGDEAQSKR	10	Par17	76	IMEAMEKLK	7
17	P09127	88	AELEGIIKQQAAQLK	8	P77187	300	GTKEAALK	3
18	P63261	313	MQKEITALAPSTMK	3	P63261	207	EIVRDIKEK	7
19	Par17	73	HGKIMEAMEK	3	Par17	76	IMEAMEKLK	7
20	P63261	313	MQKEITALAPSTMK	3	P63261	51	DSYVGDEAQSKR	11
21	Par17	53	AQGPKGGGNAVK	5	P03014	40	SERPGLK	1
22	Par17	89	FNEVAAQYSEDKAR	8	Par17	76	IMEAMEKLK	7
23	Par17	89	FNEVAAQYSEDKAR	12	Par17	76	IMEAMEKLK	7
24	P63261	40	HQGVMVGMGQKDSYVGDEAQSK	11	Par17	76	IMEAMEKLK	7
25	P63261	316	EITALAPSTMKIK	11	P63261	51	DSYVGDEAQSKR	11
26	P63261	327	IKIIAPPERK	2	Par17	76	IMEAMEKLK	7
27	P63261	96	VAPEEHPVLLTEAPLNPKANR	18	P63261	51	DSYVGDEAQSKR	10
28	Par17	19	VQQQASKMPPK	6	P03018	410	VVNTPTR	4
29	P63261	313	MQKEITALAPSTMK	3	Par17	89	FNEVAAQYSEDKAR	12
30	P63261	313	MQKEITALAPSTMK	3	P63261	327	IKIIAPPERK	2
31	P63261	40	HQGVMVGMGQKDSYVGDEAQSK	11	P63261	313	MQKEITALAPSTMK	3

32	Par17	19	VQQQASKMPPK	7	P03018	410	VVNTPTR	4
33	P63261	96	VAPEEHPVLLTEAPLNPKANREK	18	Par17	89	FNEVAAQYSEDKAR	12
34	P63261	96	VAPEEHPVLLTEAPLNPKANR	11	P63261	327	IKIIAPPERK	2
35	P63261	96	VAPEEHPVLLTEAPLNPKANREK	18	P63261	313	MQKEITALAPSTMK	3
36	P63261	313	MQKEITALAPSTMK	3	Par17	76	IMEAMEKLK	7
37	Par17	89	FNEVAAQYSEDKAR	8	Par17	143	TKFGYHIIMVEGR	2
38	P63261	327	IKIIAPPERK	2	P0AA37	89	EREPKK	5
39	P63261	96	VAPEEHPVLLTEAPLNPKANREK	18	P63261	327	IKIIAPPER	2
40	P63261	96	VAPEEHPVLLTEAPLNPKANREK	18	Par17	76	IMEAMEKLK	7
41	P63261	313	MQKEITALAPSTMK	3	P63261	316	EITALAPSTMKIK	11
42	P63261	96	VAPEEHPVLLTEAPLNPKANR	18	P63261	316	EITALAPSTMKIK	11
43	P06992	227	NSLGNLFSVEVLTGMGIDPAMR	2	Par17	89	FNEVAAQYSEDKAR	12
44	P0ADQ5	1	MNTVFLHLSEEAIK	3	P26218	170	VYNENGKR	2
45	P06992	227	NSLGNLFSVEVLTGMGIDPAMR	2	Par17	89	FNEVAAQYSEDKAR	9
46	P63261	51	DSYVGDEAQSKR	10	Par17	1	GPMAGLLKGLVR	8
47	P0ACL2	21	IEQGVYLVGDKLPAERFIADEK	11	Par17	76	IMEAMEKLK	7
48	P09323	455	AESIGDAMKKVVAR	10	P63261	207	EIVRDIKEK	7
49	Par17	89	FNEVAAQYSEDKAR	9	P76066	31	ATSIELAARLDISR	2
50	P04995	114	NIFYRNFYDPYAWSWQHDNSR	4	P63261	96	VAPEEHPVLLTEAPLNPKANR	11
51	Par17	113	GSMVGPFQEAAFALPVSGMDKPVFTDPPVK	2	Par17	19	VQQQASKMPPK	7
52	Par17	143	TKFGYHIIMVEGR	1	P0ACP7	325	LIERRSVADGPFR	6
53	Par17	113	GSMVGPFQEAAFALPVSGMDKPVFTDPPVK	21	Par17	89	FNEVAAQYSEDKAR	12
54	Par17	113	GSMVGPFQEAAFALPVSGMDKPVFTDPPVK	17	P63261	96	VAPEEHPVLLTEAPLNPKANR	18
55	P63261	178	LDLAGRDLTDYLMKILTER	14	Par17	67	HILCEKHGK	6
56	Par17	143	TKFGYHIIMVEGR	2	P39393	549	VTMPEIWKLDR	8
57	P63261	96	VAPEEHPVLLTEAPLNPKANR	11	P77239	212	APIDGVITAFDLR	8
58	Par17	113	GSMVGPFQEAAFALPVSGMDKPVFTDPPVK	21	P63261	313	MQKEITALAPSTMK	3
59	Par17	113	GSMVGPFQEAAFALPVSGMDKPVFTDPPVK	25	Par17	76	IMEAMEKLK	7
60	Par17	1	GPMAGLLKGLVR	8	P0A784	176	EHFIKL	5
61	P63261	184	DLTDYLMKILTER	8	P0A9J8	358	ALKELGEITR	3
62	P63261	96	VAPEEHPVLLTEAPLNPKANREK	11	P63261	178	LDLAGRDLTDYLMKILTER	9
63	P63261	40	HQGVMVGMGQKDSYVGDEAQSK	11	P63261	178	LDLAGRDLTDYLMKILTER	14
64	P63261	178	LDLAGRDLTDYLMKILTER	14	Par17	19	VQQQASKMPPK	7

65	P63261	178	LDLAGRDLTDYLMKILTER	14	P63261	327	IKIIAPPERK	2
66	P63261	178	LDLAGRDLTDYLMKILTER	14	P63261	197	GYSFTTTAEREIVRDIKEK	17
67	P63261	178	LDLAGRDLTDYLMKILTER	14	Par17	1	MPPKGK	4
68	Par17	89	FNEVAAQYSEDKAR	12	P63261	178	LDLAGRDLTDYLMKILTER	14
69	P63261	96	VAPEEHPVLLTEAPLNPKANREK	18	P63261	178	LDLAGRDLTDYLMKILTER	14
70	P63261	178	LDLAGRDLTDYLMKILTER	14	P63261	51	DSYVGDEAQSKR	10
71	P63261	178	LDLAGRDLTDYLMKILTER	14	P63261	51	DSYVGDEAQSKR	11
72	Par17	113	GSMVGPFQEAAFALPVSGMDKPVFTDPPVK	21	P0A9C5	104	DPRSIAK	4
73	P63261	313	MQKEITALAPSTMK	3	P63261	178	LDLAGRDLTDYLMKILTER	14
74	P63261	178	LDLAGRDLTDYLMKILTER	14	Par17	76	IMEAMEKLK	7
75	P63261	184	DLTDYLMKILTER	3	Par17	76	IMEAMEKLK	7
76	P63261	184	DLTDYLMKILTER	5	Par17	76	IMEAMEKLK	7
77	P37640	2	QIVMFDRQSIFIHGMK	9	P63261	184	DLTDYLMKILTER	8
78	P63261	316	EITALAPSTMKIK	11	P63261	178	LDLAGRDLTDYLMKILTER	14
79	Par17	113	GSMVGPFQEAAFALPVSGMDKPVFTDPPVK	25	P0AAL3	1	MSRSAKPQNGRR	6
80	Par17	113	GSMVGPFQEAAFALPVSGMDKPVFTDPPVK	21	P63261	184	DLTDYLMKILTER	3
81	Par17	53	AQGPKGGGNAVK	5	Par17	32	SGSGKAGK	5
82	P63261	51	DSYVGDEAQSKR	11	Par17	67	HILCEKHGK	6
83	Par17	89	FNEVAAQYSEDKAR	9	Par17	53	AQGPKGGGNAVK	5
84	Par17	89	FNEVAAQYSEDKAR	9	Par17	83	LKSGMR	2
85	Par17	58	GGGNAVKVR	7	Par17	76	IMEAMEKLK	7
86	P63261	316	EITALAPSTMKIK	11	Par17	76	IMEAMEKLK	7
87	P0AAF1	9	MGVVQLTILTMVNMMGSGIIMLPTK	24	P63261	207	EIVRDIKEK	7
88	P0ADT2	15	NWSARHLTPVALAVATVFMLAGCEK	8	Par17	89	FNEVAAQYSEDKAR	12
89	P63261	327	IKIIAPPER	2	P0AC41	313	LKLDHLGK	2
90	P63261	178	LDLAGRDLTDYLMKILTER	14	Par17	1	GPMAGLLKGLVR	8
91	Par17	89	FNEVAAQYSEDKAR	9	P76514	168	FNDLSELRK	5
92	Par17	89	FNEVAAQYSEDKAR	8	Par17	19	VQQQASKMPPK	7
93	Par17	143	TKFGYHIIMVEGR	2	Par17	19	VQQQASKMPPK	7
94	Par17	89	FNEVAAQYSEDKAR	9	Par17	76	IMEAMEKLK	7
95	P63261	313	MQKEITALAPSTMK	6	P63261	327	IKIIAPPERK	2
96	P06992	227	NSLGNLFSVEVLTGMGIDPAMR	2	Par17	89	FNEVAAQYSEDKAR	8
97	Par17	113	GSMVGPFQEAAFALPVSGMDKPVFTDPPVK	25	Par17	53	AQGPKGGGNAVK	5

98	Par17	113	GSMVGPFQEAAFALPVSGMDKPVFTDPPVK	17	P77509	441	QQIYR	4
99	Par17	113	GSMVGPFQEAAFALPVSGMDKPVFTDPPVK	21	Par17	83	LKSGMR	2
100	Par17	143	TKFGYHIIMVEGR	2	P0ACP7	325	LIERRSVADGPFR	6
101	Par17	113	GSMVGPFQEAAFALPVSGMDKPVFTDPPVK	25	Par17	89	FNEVAAQYSEDKAR	12
102	Par17	113	GSMVGPFQEAAFALPVSGMDKPVFTDPPVK	17	P63261	51	DSYVGDEAQSKR	11
103	Par17	113	GSMVGPFQEAAFALPVSGMDKPVFTDPPVK	21	P0A9C0	1	MKTRDSQSSDVIIIGGGATGAGIAR	2
104	P63261	178	LDLAGRDLTDYLMKILTER	14	P63261	316	EITALAPSTMKIK	8
105	P63261	184	DLTDYLMKILTER	3	P63261	327	IKIIAPPER	2
106	P63261	178	LDLAGRDLTDYLMKILTER	17	P13656	575	ERVGTPK	5
107	Par17	37	AGKGGAASGSDSADK	3	Par17	52	KAQGPK	1
108	Par17	89	FNEVAAQYSEDKAR	12	Par17	53	AQGPKGGGNAVK	5
109	Par17	89	FNEVAAQYSEDKAR	12	Par17	26	MPPKGK	4
110	Par17	89	FNEVAAQYSEDKAR	12	Par17	83	LKSGMR	2
111	Par17	76	IMEAMEKLK	7	Par17	26	MPPKGK	4
112	Par17	89	FNEVAAQYSEDKAR	12	P63261	327	IKIIAPPERK	2
113	P63261	96	VAPEEHPVLLTEAPLNPKANR	18	P63261	197	GYSFTTTAEREIVRDIKEK	17
114	Par17	1	GPMAGLLKGLVR	8	Par17	67	HILCEKHGK	6
115	P63261	96	VAPEEHPVLLTEAPLNPKANREK	18	P63261	96	VAPEEHPVLLTEAPLNPKANR	18
116	P69811	251	LADLLLDNKADR	9	Par17	67	HILCEKHGK	6
117	Par17	143	TKFGYHIIMVEGR	2	Par17	67	HILCEKHGK	6
118	Par17	1	GPMAGLLKGLVR	8	Par17	76	IMEAMEKLK	7
119	Par17	89	FNEVAAQYSEDKAR	9	Par17	143	TKFGYHIIMVEGR	2

HADDOCK restraints

Ambigious interaction restaints:

Par17 1,2,3,4,5,6,7,8,9,10,11,12,13,14,15,16,17,18,19,20,21,22,23,24,25,26,27,28,29,30,31,32,33,34,35,36,37,38,39,40,66,84,100,149

Actin all residues from spotter:

29,30,38,39,40,41,42,43,44,45,46,47,48,49,50,59,60,61,62,63,64,66,68,84,85,86,87,88,89,90,91,92,93,94,95,96,129,130,131,132,133,134,135,136,137,138,139,14[illegible]
141,142,143,144,145,146,147,148,149,150,151,152,166,167,168,169,170,171,172,173,174,175,176,177,178,179,180,189,190,191,192,193,194,195,196,197,198,
199,200,201,202,203,204,247,248,249,250,251,252,253,254,255,256,257,258,259,260,261,262,263,264,301,302,303,304,305,306,307,308,309,310,311,312,313,
314,329,330,331,332,333,334,335,336,337,338,339,340,341,342,343,344,345,346,347,348,352,353,354,355,356,357,358,359,360

Actin residues interaction and exposed to the surface:

29,30,38,39,40,41,42,43,44,45,46,47,48,49,50,59,60,61,62,63,64,66,68,84,85,86,87,88,89,90,91,92,93,94,95,96,129,130,131,137,138,139,140,141,142,143,144,14[illegible]
146,147,148,149,150,166,167,168,169,170,171,172,173,174,175,176,177,178,179,190,191,193,194,195,196,197,198,199,200,201,202,203,204,248,249,251,252,
253,255,258,259,262,263,264,304,305,306,307,308,309,310,311,312,314,329,330,331,332,333,334,335,336,337,338,341,343,345,346,348,352,353,354,355,356,
358,359,360

Unambigious interaction restaints (A=Par17; B=Actin)

Protei	Position	Atom	Protein	Position	Atom	Max distance	Minimal distance	
A	8	CA)	B	191	CA)	12	10	0
A	29	CA)	B	191	CA)	12	10	0
A	25	CA)	B	191	CA)	12	10	0
A	100	CA)	B	191	CA)	12	10	0
A	100	CA)	B	315	CA)	12	10	0
A	82	CA)	B	191	CA)	12	10	0
A	100	CA)	B	113	CA)	12	10	0
A	82	CA)	B	50	CA)	12	10	0
A	72	CA)	B	61	CA)	12	10	0
A	82	CA)	B	315	CA)	12	10	0
A	8	CA)	B	60	CA)	12	10	0
A	129	CA)	B	113	CA)	12	10	0
A	82	CA)	B	188	CA)	12	10	0
A	72	CA)	B	191	CA)	12	10	0
A	82	CA)	B	61	CA)	12	10	0
A	82	CA)	B	328	CA)	12	10	0
A	133	CA)	B	315	CA)	12	10	0
A	82	CA)	B	326	CA)	12	10	0
