# Supplementary material for: The Actin‐Binding Prolyl‐Isomerase Par17 Sustains Its Substrate Selectivity by Interdomain Allostery
Source: Proteins. 2025 Mar 12;93(9):1481–97. doi: 10.1002/prot.26807 (PMC12314576; doi:10.1002/prot.26807)
Supplement: Supplementary file 6 — Table S6. 12‐mers of β‐actin used in the peptide array, identified as possible target regions for Par14 (red), Par17 (blue), and both Par14/17 (purple). [file PROT-93-1481-s008.pdf]

**12-mers of β-actin used in the peptide array, identified as possible target regions for Par14 (red), Par17 (blue) and both Par14/17 (purple)**

| | 1 | 2 | 3 | 4 | 5 | 6 | 7 | 8 | 9 | 10 |
|---|---|---|---|---|---|---|---|---|---|---|
| A | | | | | | | | | | |
| B | | | EKIWHHTFYNEL | | | | | | | |
| C | | | IWHHTFYNELRV | | | | GQVITIGNERFR | | | |
| D | | | HHTFYNELRVAP | FNTPAMYVAIQA | EGYALPHAILRL | | VITIGNERFRCP | | IKIIAPPERKYS | |
| E | | | TFYNELRVAPEE | TPAMYVAIQAVL | YALPHAILRLDL | | TIGNERFRCPEA | | IIAPPERKYSVW | |
| F | | | | AMYVAIQAVLSL | LPHAILRLDLAG | | GNERFRCPEALF | | APPERKYSVWIG | |
| G | | YVGDEAQSKRGI | | YVAIQAVLSLYA | | KEKLCYVALDFE | ERFRCPEALFQP | | PERKYSVWIGGS | |
| H | | GDEAQSKRGILT | | AIQAVLSLYASG | | KLCYVALDFEQE | FRCPEALFQPSF | | RKYSVWIGGSIL | |
| I | | EAQSKRGILTLK | | QAVLSLYASGRT | | CYVALDFEQEMA | | | YSVWIGGSILAS | |
| J | | QSKRGILTLKYP | | VLSLYASGRTTG | | | | LSGGTTMYPGIA | VWIGGSILASLS | |
| K | | | | SLYASGRTTGIV | | | | GGTTMYPGIADR | | |
| L | | | | YASGRTTGIVMD | | | | TTMYPGIADRMQ | | |
| M | | | | | | | | MYPGIADRMQKE | ILASLSTFQQMW | |
| N | PRAVFPSIVGRP | | | | DYLMKILTERGY | | | | ASLSTFQQMWIS | |
| O | AVFPSIVGRPRH | | | | LMKILTERGYSF | | | | LSTFQQMWISKQ | |
| P | FPSIVGRPRHQG | | | | KILTERGYSFTT | | | | TFQQMWISKQEY | |
| Q | SIVGRPRHQGVM | | | | LTERGYSFTTTA | | | | | |
| R | VGRPRHQGVMVG | | | | ERGYSFTTTAER | | | | | |
| S | RPRHQGVMVGMG | | | | | | | | | |
| T | RHQGVMVGMGQK | | | | | | | | | |

**sequence of β-actin, the 12mers used in the peptide array are highlighted in yellow**

**β-Actin**

```
        10         20         30         40         50         60
MDDDIAALVV DNGSGMCKAG FAGDDAPRAV FPSIVGRPRH QGVMVGMGQK DSYVGDEAQS

        70         80         90        100        110        120
KRGILTLKYP IEHGIVTNWD DMEKIWHHTF YNELRVAPEE HPVLLTEAPL NPKANREKMT

       130        140        150        160        170        180
QIMFETFNTP AMYVAIQAVL SLYASGRTTG IVMDSGDGVT HTVPIYEGYA LPHAILRLDL

       190        200        210        220        230        240
AGRDLTDYLM KILTERGYSF TTTAEREIVR DIKEKLCYVA LDFEQEMATA ASSSSLEKSY

       250        260        270        280        290        300
ELPDGQVITI GNERFRCPEA LFQPSFLGME SCGIHETTFN SIMKCDVDIR KDLYANTVLS

       310        320        330        340        350        360
GGTTMYPGIA DRMQKEITAL APSTMKIKII APPERKYSVW IGGSILASLS TFQQMWISKQ

       370
EYDESGPSIV HRKCF
```
