# Supplementary material for: The Actin‐Binding Prolyl‐Isomerase Par17 Sustains Its Substrate Selectivity by Interdomain Allostery
Source: Proteins. 2025 Mar 12;93(9):1481–97. doi: 10.1002/prot.26807 (PMC12314576; doi:10.1002/prot.26807)
Supplement: Supplementary file 7 — Table S7. Intramolecular cross‐linking of hPar14/17 with genetically coded photo amino acids. [file PROT-93-1481-s003.pdf]

Intramolecular cross-linking of hPar14/17 with genetically coded photo amino acids

Irradiated hPar14 Photo-Met-Leu (Monomer)
Irradiated hPar17 Photo-Met-Leu (Monomer)

LC-settings:

LC device	Thermo Easy-nLC 1000
**Analytical column**	Self-packed fused silica capillary with integrated pico frit emitter; New Objectives PF360-75-15-N-5
column diameter	Length (L_C) = 35 cm; ID = 75µm; OD = 360 µm; emitter 15 µm
stationary phase	Reprosil-Pur 120 C18-AQ, Dr. Maisch GmbH
particle diameter (d_p)	1.9 µm
Pore size	120 Å
Column ID	AC62
Column oven	Sonation column oven PRSO-V1
Column oven temp.	45°C
**Analytical column**	Self-packed fused silica capillary with integrated pico frit emitter; New Objectives PF360-75-15-N-5
column diameter	Length (L_C) = 35 cm; ID = 75µm; OD = 360 µm; emitter 15 µm
stationary phase	Reprosil-Pur 120 C18-AQ, Dr. Maisch GmbH
**solvents**	A: 0.1% FA in UPLC water<br>B: 0.1% FA in 80% UPLC ACN
gradient	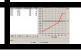

MS-settings:

**MS**	**general**	**MS1**	**MS2**	**MS2**	**MS3**	**Comments; special settings**
Elite	TunePlus v2.7.0 SP1<br>Gradient: 70 min	Analyzer: FT<br>Res.: 60000<br>SR: 350 - 1500<br>AGC: 1×10^6<br>AcT: 50<br>DDM: NS	Analyzer: FT<br>Res.: 15000<br>SR: variable<br>AGC: 1×10^5<br>AcT: 120 ms<br>CS: >+2<br>Frag.: CID<br>NCE: 35<br>NS: 5×	Analyzer: FT<br>Res.: 15000<br>SR: variable<br>AGC: 1×10^5<br>AcT: 120 ms<br>CS: >+2<br>Frag.: ETD<br>NCE: n/a<br>NS: 5×		5th order double play. MS2 was done in OT. Each mass selected for fragmentation was first fragmented by HCD and then by ETD. MS2's were only performed on charge states >+2.

Note: **FT**= Fourier Transform (Orbitrap); **IT**= Iontrap; **Q**= Quadrupol; **Res.**= max. Resolution at 200 m/z (Lumos) or 400 m/z (Elite) [FWHM (full width at half maximum)]; **ScR**= scan rate for measurements in the IT; **SR**= scan range [m/z]; **AGC**= automatic gain control, max number of acquired ions per measurement; **AcT**= max. Ion acquisition time [ms]; **CS**= charge states used for fragmentation; **IsM**= Isolation mode (Q or IT), MS2 isolation and further is only done in IT; **IsW**= Isolation window [m/z], value followed by scan mode the isolation is based on (MS1, MS2 …) **Frag.**= Fragmentation method; **HCD**= Higher-energy collisional dissociation; **CID**= Collision-induced dissociation; **ETD**= Electron-transfer dissociation**; EThcD=** Electron-Transfer/Higher-Energy Collision Dissociation; **sHCD**= stepped HCD**; NCE**= normalized collision energy; **cycles**: number of MSn recorded or max cycle time; RF= RF Lens [%]; **SF**= Source Fragmentation [V]; **DDM**: Data dependent Mode (cycle time in seconds, CT/[s] or number of scans, NS); **NS**= Number of data dependent scans

Search-settings:

Program & version	StavroX v3.6.6
Search engine	StavroX v3.6.6
settings	AG01_hPar14ML_settings_v05.zhrs<br>AG04_hPar17ML_settings_v05.zhrs
Static modification	none
Digestion mode	Trypsin/P (specific), 3 missed cleavages
Dynamic modification	Carbamidomethyl (C)
Modification included in quantification	No quantification
Databases	AG01: Par14ML.fasta<br>AG04: hPar17ML.fasta
Annotation	

Intramolecular cross linking of hPar14/17 with DSSO

hPar14 (monomeric) crosslinked by DSSO
hPar17 (monomeric) crosslinked by DSSO

LC-settings:

LC device	Thermo Easy-nLC 1000
**Analytical column**	Self-packed fused silica capillary with integrated pico frit emitter; New Objectives PF360-75-15-N-5
column diameter	Length (L_C) = 37 cm; ID = 75µm; OD = 360 µm; emitter 15 µm
stationary phase	Reprosil-Pur 120 C18-AQ, Dr. Maisch GmbH
particle diameter (d_p)	1.9 µm
Pore size	120 Å
Column ID	AC65
Column oven	Sonation column oven PRSO-V1
Column oven temp.	45°C
**solvents**	A: 0.1% FA in UPLC water<br>B: 0.1% FA in 80% UPLC ACN
gradient	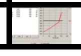

MS-settings:

**MS**	**general**	**MS1**	**MS2**	**MS2**	**MS3**	**Comments; special settings**
Elite	TunePlus v2.7.0 SP1<br>Gradient: 160 min	Analyzer: FT<br>Res.: 120000<br>SR: 300 - 2000<br>AGC: $1×10^6$<br>AcT: 70<br>DDM: NS	Analyzer: FT<br>Res.: 15000<br>SR: variable<br>AGC: $5×10^5$<br>AcT: 250 ms<br>CS: >+2<br>Frag.: HCD<br>NCE: 31<br>NS: 10×			MS2 was done in OT; MS2 was performed only on charge states >+2.

Note: **FT**= Fourier Transform (Orbitrap); **IT**= Iontrap; **Q**= Quadrupol; **Res.**= max. Resolution at 200 m/z (Lumos) or 400 m/z (Elite) [FWHM (full width at half maximum)]; **ScR**= scan rate for measurements in the IT; **SR**= scan range [m/z]; **AGC**= automatic gain control, max number of acquired ions per measurement; **AcT**= max. Ion acquisition time [ms]; **CS**= charge states used for fragmentation; **IsM**= Isolation mode (Q or IT), MS2 isolation and further is only done in IT; **IsW**= Isolation window [m/z], value followed by scan mode the isolation is based on (MS1, MS2 …) **Frag.**= Fragmentation method; **HCD**= Higher-energy collisional dissociation; **CID**= Collision-induced dissociation; **ETD**= Electron-transfer dissociation**; EThcD=** Electron-Transfer/Higher-Energy Collision Dissociation; **sHCD=** stepped HCD**; NCE**= normalized collision energy; **cycles**: number of MSn recorded or max cycle time; RF= RF Lens [%]; **SF**= Source Fragmentation [V]; **DDM**: Data dependent Mode (cycle time in seconds, CT/[s] or number of scans, NS); **NS**= Number of data dependent scans

Search-settings:

Program & version	ProteomeDscoverer 2.2. or PD2.4
Search engine	Sequest and XlinkX
settings	See there
Static modification	Carbamidomethyl (C)
Digestion mode	Trypsin/P (specific), 2 missed cleavages
Dynamic modification	Acetyl (N-term); Oxidation (M); DSSO; DSSO Amidated; DSSO hydrolyzed
Modification included in quantification	Oxidation (M)
Databases	Contaminants<br>AG01: ACE_0403_UP000000625_83333_plus_hPar14-WT_v01.fasta<br>AG02: ACE_0403_UP000000625_83333_plus_hPar17-WT_v01.fasta
Annotation	

Crosslinking of hPar14/17 with Actin by DSSO

25 µM hPar17 and 10 µM Actin crosslinked by DSSO

LC-settings:

LC device	Thermo Easy-nLC 1200
**Analytical column**	Self-packed fused silica capillary with integrated pico frit emitter; New Objectives PF360-75-15-N-5
column diameter	Length (L_C) = 45 cm; ID = 75µm; OD = 360 µm; emitter 15 µm
stationary phase	Reprosil-Pur 120 C18-AQ, Dr. Maisch GmbH
particle diameter (d_p)	1.9 µm
Pore size	120 Å
Column ID	AC72
Column oven	Sonation column oven PRSO-V2
Column oven temp.	50°C
**solvents**	A: 0.1% FA in UPLC water<br>B: 0.1% FA in 80% UPLC ACN
gradient	

MS-settings:

**MS**	**general**	**MS1**	**MS2**	**MS2**	**MS3**	**Comments; special settings**
Lumos	Tune v3.1.2412.25	Analyzer: FT	Analyzer: FT	Analyzer: FT	Analyzer: IT	First MS2 is triggered by targeted mass difference 31.9721 (DSSO). Second MS2 is performed in OT with EThcD. Fragments from first MS2 with triggered mass difference were collected for MS3 experiments (HCD in IT). The whole sequence MS1-MS2-MS2/MS3 was repeated for 5 sec (cycle time between master scans).
	Gradient: 105 min	Res.: 120000	Res.: 30000	Res.: 50000	Res.: 15000	
		SR: 375 - 1600	SR: Auto	SR: Auto	SR: Auto	
		AGC: standard	AGC: Auto	AGC: 400%	AGC: 200%	
		AcT: 50	AcT: 100 ms	AcT: 150 ms	AcT: 120 ms	
		RF: 30	CS: >+2	CS: >+2	CS: >+2	
		SF: 10V	IsM: Q	IsM: Q	IsM: IT	
		DDM: CT/5sec	IsW: 1.6 (MS)	IsW: 1.6 (MS)	IsW: 2.5 (MS)	
			Frag.: CID<br>NCE: 25	Frag.: EThcD<br>NCE: 20<br>NS: 1	2 (MS2)<br>Frag.: HCD<br>NCE: 35<br>NS: 4	

Note: **FT**= Fourier Transform (Orbitrap); **IT**= Iontrap; **Q**= Quadrupol; **Res.**= max. Resolution at 200 m/z (Lumos) or 400 m/z (Elite) [FWHM (full width at half maximum)]; **ScR**= scan rate for measurements in the IT; **SR**= scan range [m/z]; **AGC**= automatic gain control, max number of acquired ions per measurement; **AcT**= max. Ion acquisition time [ms]; **CS**= charge states used for fragmentation; **IsM**= Isolation mode (Q or IT), MS2 isolation and further is only done in IT; **IsW**= Isolation window [m/z], value followed by scan mode the isolation is based on (MS1, MS2 …) **Frag.**= Fragmentation method; **HCD**= Higher-energy collisional dissociation; **CID**= Collision-induced dissociation; **ETD**= Electron-transfer dissociation**; EThcD=** Electron-Transfer/Higher-Energy Collision Dissociation; **sHCD**= stepped HCD**; NCE**= normalized collision energy; **cycles**: number of MSn recorded or max cycle time; RF= RF Lens [%]; **SF**= Source Fragmentation [V]; **DDM**: Data dependent Mode (cycle time in seconds, CT/[s] or number of scans, NS); **NS**= Number of data dependent scans

Search-settings:

Program & version	PD 2.2 or PD2.4.
Search engine	XlinkX 2.0 (for cross links)
settings	Basically default
Static modification	Carbamidomethyl (C)
Digestion mode	Trypsin/P (specific), 2 missed cleavages, 10ppm, 0.02 ppm
Dynamic modification	Oxidation (M), DSSO Amidated, DSSO Hydrolyzed, DSSO Tris
Modification included in quantification	Oxidation (M)
Databases	1. ACE_0439_SOI_v01.fasta (XlinkX)
Annotation	
