# Supplementary material for: The Actin‐Binding Prolyl‐Isomerase Par17 Sustains Its Substrate Selectivity by Interdomain Allostery
Source: Proteins. 2025 Mar 12;93(9):1481–97. doi: 10.1002/prot.26807 (PMC12314576; doi:10.1002/prot.26807)
Supplement: Supplementary file 8 — Table S8. Intermolecular hydrogen bonds within the Haddock hPar17‐Actin complex calculated by YASARA. [file PROT-93-1481-s005.pdf]

Supplementary Table ST8: Intermolecular hydrogen bonds within the Haddock hPar17-Actin complex calculated by YASARA.

**Hydrogen bonds**	
**hPar17**	**β-Actin**
K8	C272
Q13	Q263
K72	S199
E78	R39
K82	T203
R88	V76
E91	T77
Q95	E72
Q95	D187
Y96	T201
E98	K191
K100	L267
K100	G268
K100	E270
R102	D184
R102	A260
Q103	E270
