# Supplementary material for: The Actin‐Binding Prolyl‐Isomerase Par17 Sustains Its Substrate Selectivity by Interdomain Allostery
Source: Proteins. 2025 Mar 12;93(9):1481–97. doi: 10.1002/prot.26807 (PMC12314576; doi:10.1002/prot.26807)
Supplement: Supplementary file 9 — Table S9. Residues found to be mutated in β‐actin. [file PROT-93-1481-s001.pdf]

Supplementary Table ST9: Residues found to be mutated in β-actin. Mutations are depicted either from the COSMIC data bank of somatic mutations or from (Verloes at al., 2015; Parker et al., 2020). (X) BWS, residue mutated to residue (X) causes Baraitser-Wilson Syndrom (BWS). HB, hydrogen bond to hPar17. Color code is set according to **supplementary figure S6** (red: D-loop; yellow: sensor loop; green: helix 5; blue: Threonine-rich region; magenta: hydrophobic plug).

Mutated residues	Annotations
R39	HB
M44	
P70	
H73	(V) BWS; detects nucleotide state
G71	(S) BWS
I72	(T) BWS
V76	HB
T77	HB
R183	(W) BWS
T186	
Y188	
R196	(C,H) BWS
G197	
F200	
T201	
A204	(G) BWS
E205	
A260	
S265	
G268	HB; (R) BWS; contact to H40 ensures actin polymerisation (inner filament contact)
E270	
