# Supplementary material for: The Actin‐Binding Prolyl‐Isomerase Par17 Sustains Its Substrate Selectivity by Interdomain Allostery
Source: Proteins. 2025 Mar 12;93(9):1481–97. doi: 10.1002/prot.26807 (PMC12314576; doi:10.1002/prot.26807)
Supplement: Supplementary file 10 — Table S10. HADDOCK statistics for the most reliable cluster (cluster 1). [file PROT-93-1481-s002.pdf]

HADDOCK statistics for the most reliable cluster (cluster 1).

HADDOCK score	652.7 ± 8.2
Cluster size	70
RMSD from the overall lowest-energy structure	10.5 ± 0.1
Van der Waals energy	-83.1 ± 2.9
Electrostatic energy	-429.0 ± 20.8
Desolvation energy	15.3 ± 2.5
Restraints violation energy	8062.9 ± 103.5
Buried Surface Area	2928.5 ± 40.0
Z-score	-1.0
