# Supplementary material for: The Actin‐Binding Prolyl‐Isomerase Par17 Sustains Its Substrate Selectivity by Interdomain Allostery
Source: Proteins. 2025 Mar 12;93(9):1481–97. doi: 10.1002/prot.26807 (PMC12314576; doi:10.1002/prot.26807)
Supplement: Supplementary file 11 — Table S11. YASARA Console. [file PROT-93-1481-s006.pdf]

YASARA CONSOLE

Obj	Number	Class	Atom/Residue 1				Atom/Residue 2				d	d-	d+	Scale	dNow	dViol	E[kJ/mol]
1	1	-	NZ	LYS	8	A-	NZ	LYS	191	B	10.100	3.000	3.000	1.0	13.866	0.766	2.456
1	2	-	NZ	LYS	29	A-	NZ	LYS	191	B	10.100	3.000	3.000	1.0	12.892		0.000
1	3	-	NZ	LYS	25	A-	NZ	LYS	191	B	10.100	3.000	3.000	1.0	11.812		0.000
1	4	-	NZ	LYS	100	A-	NZ	LYS	191	B	10.100	3.000	3.000	1.0	9.375		0.000
1	5	-	NZ	LYS	100	A-	NZ	LYS	315	B	10.100	3.000	3.000	1.0	19.766	6.666	51.596
1	6	-	NZ	LYS	82	A-	NZ	LYS	191	B	10.100	3.000	3.000	1.0	16.239	3.139	22.087
1	7	-	NZ	LYS	100	A-	NZ	LYS	113	B	10.100	3.000	3.000	1.0	20.362	7.262	56.582
1	8	-	NZ	LYS	82	A-	NZ	LYS	50	B	10.100	3.000	3.000	1.0	13.646	0.547	1.250
1	9	-	NZ	LYS	72	A-	NZ	LYS	61	B	10.100	3.000	3.000	1.0	17.915	4.815	36.111
1	10	-	NZ	LYS	82	A-	NZ	LYS	315	B	10.100	3.000	3.000	1.0	28.562	15.462	125.205
1	11	-	NZ	LYS	8	A-	CB	SER	60	B	10.100	3.000	3.000	1.0	33.676	20.576	167.996
1	12	-	CB	SER	129	A-	NZ	LYS	113	B	10.100	3.000	3.000	1.0	12.422		0.000
1	13	-	NZ	LYS	82	A-	CB	TYR	188	B	10.100	3.000	3.000	1.0	14.007	0.907	3.441
1	14	-	NZ	LYS	72	A-	NZ	LYS	191	B	10.100	3.000	3.000	1.0	13.489	0.390	0.635
1	15	-	NZ	LYS	82	A-	NZ	LYS	61	B	10.100	3.000	3.000	1.0	13.507	0.407	0.693
1	16	-	NZ	LYS	82	A-	NZ	LYS	328	B	10.100	3.000	3.000	1.0	35.787	22.687	185.658
1	17	-	NZ	LYS	133	A-	NZ	LYS	315	B	10.100	3.000	3.000	1.0	37.701	24.601	201.676
1	18	-	NZ	LYS	82	A-	NZ	LYS	326	B	10.100	3.000	3.000	1.0	37.295	24.196	198.284

Of 18 distance restraints, 14 (77.78%) are violated with a total energy of 1053.670 kJ/mol
